# MsAREB1 enhances combined cold and saline–alkali stress tolerance by promoting ascorbic acid biosynthesis in alfalfa

**DOI:** 10.1111/pbi.70156

**Published:** 2025-05-26

**Authors:** Weileng Guo, Yuanqing Sun, Juqi Chai, Lei Liu, Jiaqi Li, Yuekun Ren, Changhong Guo

**Affiliations:** ^1^ Key Laboratory of Molecular and Cytogenetics, College of Life Science and Technology Harbin Normal University Harbin China

**Keywords:** alfalfa, ascorbic acid, combined cold and saline–alkali stress, MsAREB1, MsILR3, MsMIOX2

## Abstract

Cold and saline–alkali stress are typical abiotic stresses on forage in middle and high latitudes, and they frequently occur simultaneously, decreasing the yield and quality of forage. Ascorbic acid plays an essential role in reactive oxygen species metabolism in response to abiotic stress. However, the molecular mechanisms of ascorbic acid biosynthesis induced by combined cold and saline–alkali stress remain unclear. This study identified an abscisic acid‐responsive element‐binding protein/ABRE‐binding factors transcription factor (TF), MsAREB1, which was significantly induced by the combined stress and abscisic acid treatment. Under combined stress, *MsAREB1* overexpression regulated ascorbic acid biosynthesis and played a role in the defence response to combined stress by positively regulating *myo‐inositol oxygenase 2* (*MsMIOX2*) expression. *MsAREB1* and *MsMIOX2* overexpression improved resistance to combined stress by increasing the ascorbic acid content. In addition, MsILR3, a bHLH TF, interacted with MsAREB1 to form a protein complex, thereby reducing the MsAREB1‐induced transcriptional activation of *MsMIOX2*. This study demonstrates a model for the regulatory mechanism of MsAREB1‐mediated regulation of *MsMIOX2* expression and ascorbic acid biosynthesis to reduce oxidative damage by combined cold and saline–alkali stress. These results provide insights for improving the resistance of plants to combined cold and saline–alkali stress and lay the foundation for the genetic improvement of stress tolerance in alfalfa.

## Introduction

Alfalfa is the most widely cultivated perennial forage legume grass worldwide, with a high grass yield, rich nutritional value, well‐developed root system and strong nitrogen‐fixing ability (Lv *et al*., 2023; Shi *et al*., 2024). Alfalfa growth in the middle and high latitudes is affected by the cold environment in winter (Zhao *et al*., 2023). Furthermore, the absorption of water and nutrients by alfalfa is severely restricted with increasing soil salinisation (Guo *et al*., 2024). However, environmental stressors usually occur simultaneously or consecutively (Gong *et al*., 2020). In saline–alkali soils of cold regions, combined cold and saline–alkali (CSA) stress severely limits alfalfa yield and quality.

Stress in plants causes the accumulation of large amounts of reactive oxygen species (ROS) such as hydrogen peroxide (H_2_O_2_), superoxide anions ($O_{2}^{-}$) and hydroxyl radicals (Chen *et al*., 2024; Liu *et al*., 2023b; Zhang *et al*., 2023). Excessive ROS usually results in oxidative stress in plants, leading to membrane dysfunction and cell death, thereby inhibiting growth and reducing yield. Ascorbic acid (AsA) is an essential reductive substance in plants that plays a pivotal role in maintaining protein stability, structural integrity of the biofilm system and defence against membrane lipid peroxidation (Yu *et al*., 2024). AsA and antioxidant enzymes such as ascorbate peroxidase, dehydroascorbic acid reductase, monodehydroascorbic acid reductase, glutathione reductase, mitochondrial reductase and other non‐enzymatic antioxidant substances, such as glutathione (GSH) and nicotinamide adenine dinucleotide phosphate, form the AsA–GSH cycle, which functions to scavenge free radicals. When plants are under CSA stress, members of the AsA–GSH cycle collaborate to convert ROS into non‐toxic substances, thereby maintaining the redox balance in cells (Liu *et al*., 2023b; Singh *et al*., 2024). However, the relationship between AsA synthesis and plant tolerance to CSA stress remains unknown.

AsA synthesis in higher species involves four pathways: D‐mannose/L‐galactose, D‐galacturonic acid, L‐glucose and myo‐inositol (MI) (Liu *et al*., 2023b; Singh *et al*., 2024). The MI pathway plays a pivotal role in AsA synthesis, which can be divided into two processes: myo‐inositol‐1‐phosphate synthase and MI monophosphatase mediate MI synthesis, and MI oxygenase (MIOX) uses synthesised MI to mediate AsA synthesis (Chen *et al*., 2023; Lisko *et al*., 2013). *PeMIPS1*, *SlIMP3*, *AtMIOX4* and *SlMIOX4* overexpression markedly increases AsA accumulation in transgenic plants (Lisko *et al*., 2013; Munir *et al*., 2020; Zhang *et al*., 2018; Zheng *et al*., 2022). However, how alfalfa synthesises AsA to eliminate ROS and respond to stress remains unclear.

Transcription factors (TFs) play a pivotal role in linking external environmental stress responses by transmitting stress signals to downstream genes. Several TFs are involved in AsA biosynthesis. For example, in kiwifruit (*Actinidia eriantha* Benth.), AceMYBS1 and AceGBF3 interact to regulate AsA content by activating GDP‐L‐galactose‐phosphorylase (*AceGGP3*) transcription (Liu *et al*., 2022). AcePosF21, a bZIP TF, mediates *AceGGP3*–AsA synthesis in kiwifruit and plays a positive regulatory role in cold stress (Liu *et al*., 2023b). Similarly, SlEIL2 is involved in AsA biosynthesis in tomatoes by regulating *SlMIOX1* and *SlGPP3*/*IMP3* expression (Chen *et al*., 2023). Studies have demonstrated a link between abscisic acid (ABA), an essential phytohormone in stress response, and AsA. Ptp‐like nucleotidase (PTPN), which is activated by the ABA signalling pathway, is a key regulator that promotes AsA production and positively regulates drought tolerance in *Arabidopsis* and maize (Zhang *et al*., 2020). In kiwifruit, ABA inhibits AsA synthesis by suppressing *AceMYBS1* expression (Liu *et al*., 2022). However, the interaction between ABA and AsA signalling pathways in the alfalfa stress response remains unclear.

Among TFs, ABA‐responsive element‐binding protein (AREBs)/ABRE‐binding factors (ABFs) play a key role in the network regulation of ABA‐related genes in response to multiple stresses in plants. *AtABF3* overexpression enhances tolerance to oxidative, salt and drought stresses in alfalfa (Wang *et al*., 2016). PpABF3 directly regulates the *PpWRKY44* promoter to promote malic acid accumulation in pears under salt stress (Ye *et al*., 2024). Under cold stress, ScAREB4 increases the trehalose content and promotes ROS scavenging by regulating *ScTPS9* and *ScGSTU8* expression (Liu *et al*., 2023a). Under low‐temperature stress, SlAREB1 affects anthocyanin biosynthesis by regulating the *SlDFR* and *SlF3′5′H* promoters through an ABA‐dependent pathway (Xu *et al*., 2023). In trifoliate orange, a transcriptional cascade comprising the TFs ABF4 and ABR1 synergistically upregulates *BAM3* expression and starch catabolism under drought stress (Zhang *et al*., 2023). However, the molecular network of the ABA signalling pathway in plants in response to CSA stress remains unknown.

This study aimed to investigate the role of MsAREB1 in regulating AsA biosynthesis in alfalfa under CSA stress. Under CSA stress, MsAREB1 directly positively regulates the transcription of *MsMIOX2* and promotes the accumulation of AsA. In addition, MsAREB1 also interacts antagonistically with the bHLH transcription factor, MsILR3, to participate in AsA synthesis. These results provide evidence that AsA alleviates oxidative damage in plants under CSA stress.

## Results

### Molecular *MsAREB1* identification and analysis

**Figure 1:**
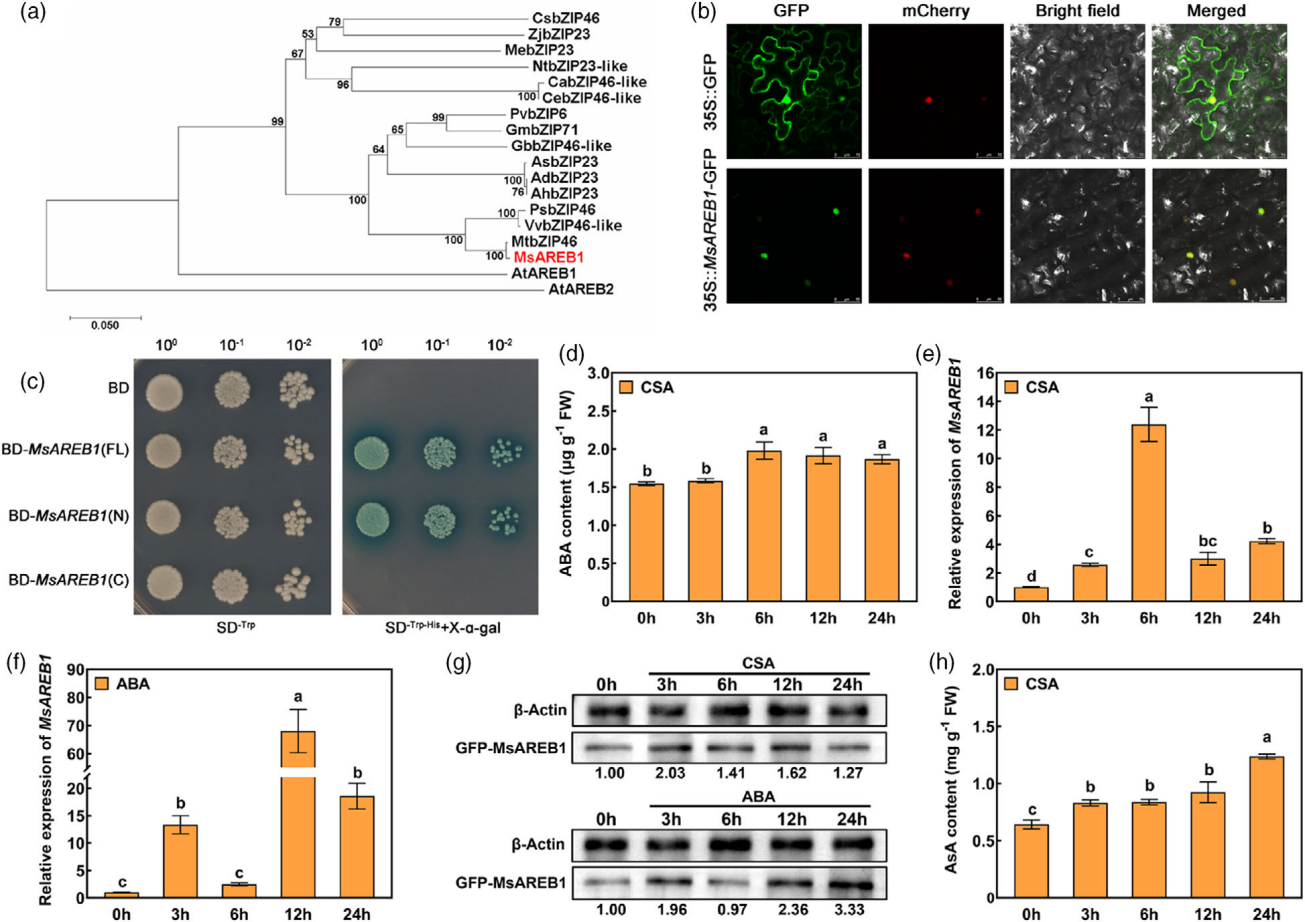
Characterisation pattern of MsAREB1 and changes in abscisic acid (ABA) and ascorbic acid (AsA) content under combined cold and saline‐alkali (CSA) stress. (a) Phylogenetic analysis of MsAREB1 and its homologues from other plant species using MEGA11 with the neighbour‐joining method and 1000 bootstraps. (b) Subcellular localisation of the 35S::MsAREB1‐GFP fusion protein in Nicotiana benthamiana leaf epidermal cells. The mCherry protein indicates nucleus localisation. Scale bars: 50 μm. (c) pGBKT7‐MsAREB1 (FL), pGBKT7‐MsAREB1 (N), and pGBKT7‐MsAREB1 (C) constructs transformed into yeast Y2HGold strain for transcriptional activation analysis. (d) ABA content in alfalfa under CSA stress. (e) MsAREB1 expression in alfalfa under CSA stress. (f) MsAREB1 expression in alfalfa under ABA treatment. (g) MsAREB1 protein abundance under CSA stress and ABA treatment. (h) AsA content in alfalfa under CSA stress. Data are mean ± SE (*n* = 3). Bars with different letters indicate a significant difference at *P* < 0.05.

The expression of differentially expressed AREB/ABF TFs revealed that MS.gene012975.t1 expression was significantly upregulated (Figure S1). MS.gene012975.t1 was annotated as *MsAREB1*. Subsequently, the open reading frame (ORF) of MS.gene012975.t1 was cloned from alfalfa (Zhaodong), totalling 1308 bp and encoding an AREB/ABF protein comprising 435 amino acids. MsAREB1 contains a basic BRLZ structural domain (Figure S2). Phylogenetic analysis indicated that it has high protein sequence homology with AtAREB1 and MtbZIP46 (Figure 1a). Subcellular localisation showed that MsAREB1 was localised in the nucleus (Figure 1b). Analysis of the yeast two‐hybrid (Y2H) assay showed that MsAREB1 possessed transcriptional activation activity. The active region was located at the N‐terminus (Figure 1c).

### CSA stress significantly induces *MsAREB1* expression and increases ABA and AsA content in alfalfa

ABA content significantly increased under CSA stress (Figure 1d). *MsAREB1* expression was significantly upregulated under CSA stress, peaking at 6 h (Figure 1e). These results indicate that CSA triggers ABA biosynthesis and signal transduction in alfalfa.

In alfalfa plants treated with ABA under CSA stress, *MsAREB1* expression was significantly elevated (Figure 1f), suggesting that ABA plays a pivotal role in CSA stress‐induced *MsAREB1* expression. Meanwhile, immunoblot analysis showed that MsAREB1 protein abundance gradually increased under CSA stress and ABA treatment in MsAREB1‐green fluorescent protein (GFP) transgenic plants (Figure 1g). In addition, CSA significantly increased the AsA content (Figure 1h). These results indicate that changes in alfalfa AsA content are associated with ABA synthesis and signalling under CSA stress and that MsAREB1 may mediate these associations.

### *MsAREB1* attenuates ROS damage and increases AsA synthesis under CSA stress

**Figure 2:**
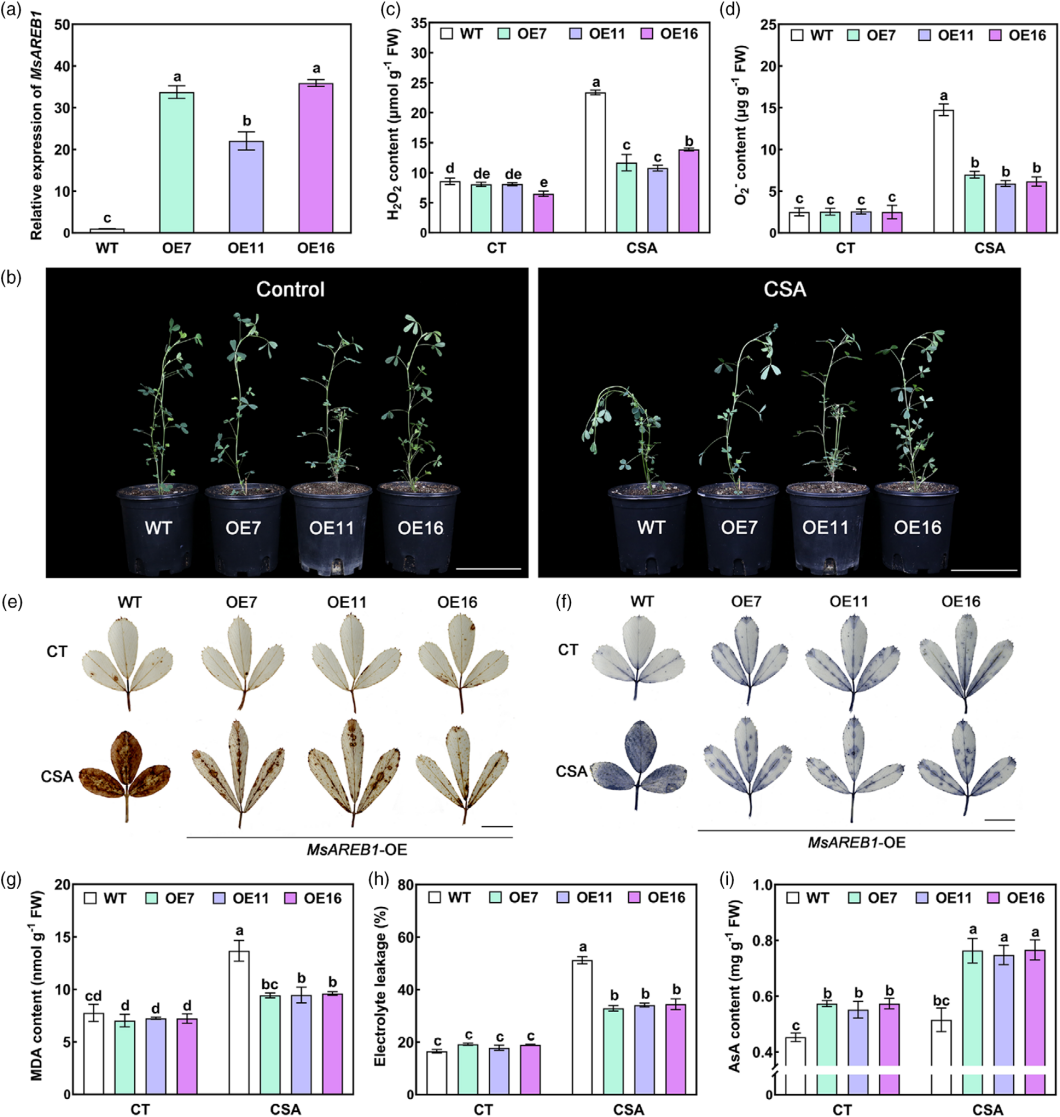
MsAREB1 positively regulates CSA stress tolerance. (a) The MsAREB1 expression in wild type (WT) and MsAREB1‐OE alfalfa. (b) Phenotypes of WT and MsAREB1‐OE alfalfa before CSA stress (Control, CT) and after CSA stress for 5 days (CSA) treatment. Scale bars: 10 cm. (c, d) Determination of hydrogen peroxide (H_2_O_2_) and superoxide anion ($O_{2}^{-}$) content. (e, f) 3,3′‐diaminobenzidine (DAB) and nitro blue tetrazolium (NBT) staining for visualising the accumulation of ROS. Scale bars: 1 cm. (g–i) Determination of malondialdehyde (MDA) content, electrolyte leakage and AsA content. Data are mean ± SE (*n* = 3). Bars with different letters indicate a significant difference at *P* < 0.05.

*MsAREB1* overexpressing transgenic alfalfa was generated to determine the function of MsAREB1 under CSA stress. Three *MsAREB1*‐overexpression (OE) lines were selected for analysis (OE7, OE11 and OE16) (Figure 2a and Figure S3). Wild‐type (WT) and *MsAREB1*‐OE lines were subjected to CSA stress. Under normal growth conditions, no significant differences were observed between the WT and *MsAREB1*‐OE lines. However, CSA stress caused severe apical bending, leaf wilting and necrosis in the WT plants, whereas no significant damage was observed in *MsAREB1*‐OE lines (Figure 2b).

*MsAREB1*‐OE lines under CSA stress had significantly lower H_2_O_2_ and O_2_^−^ contents (Figure 2c,d) and lighter 3,3′‐diaminobenzidine (DAB) and nitro blue tetrazolium (NBT) staining in leaves than the WT (Figure 2e,f). The malonaldehyde (MDA) content and electrolyte leakage were lower in alfalfa overexpressing *MsAREB1* under CSA stress than in the WT (Figure 2g,h). These results indicate that MsMIOX2 positively regulates ROS damage in alfalfa under CSA stress. As a valuable substance in the ROS detoxification process, we measured the AsA content. Under control conditions, the AsA content of *MsAREB1*‐OE lines was significantly higher than that of the WT plants. AsA contents were elevated after CSA stress, and the degree of elevation of AsA content was more significant in *MsAREB1*‐OE lines than in the WT plants (Figure 2i). These results indicate that MsAREB1 might play an important role in ROS elimination and AsA synthesis under CSA stress.

### *MsAREB1* overexpression upregulates the expression of the AsA biosynthesis gene

**Figure 3:**
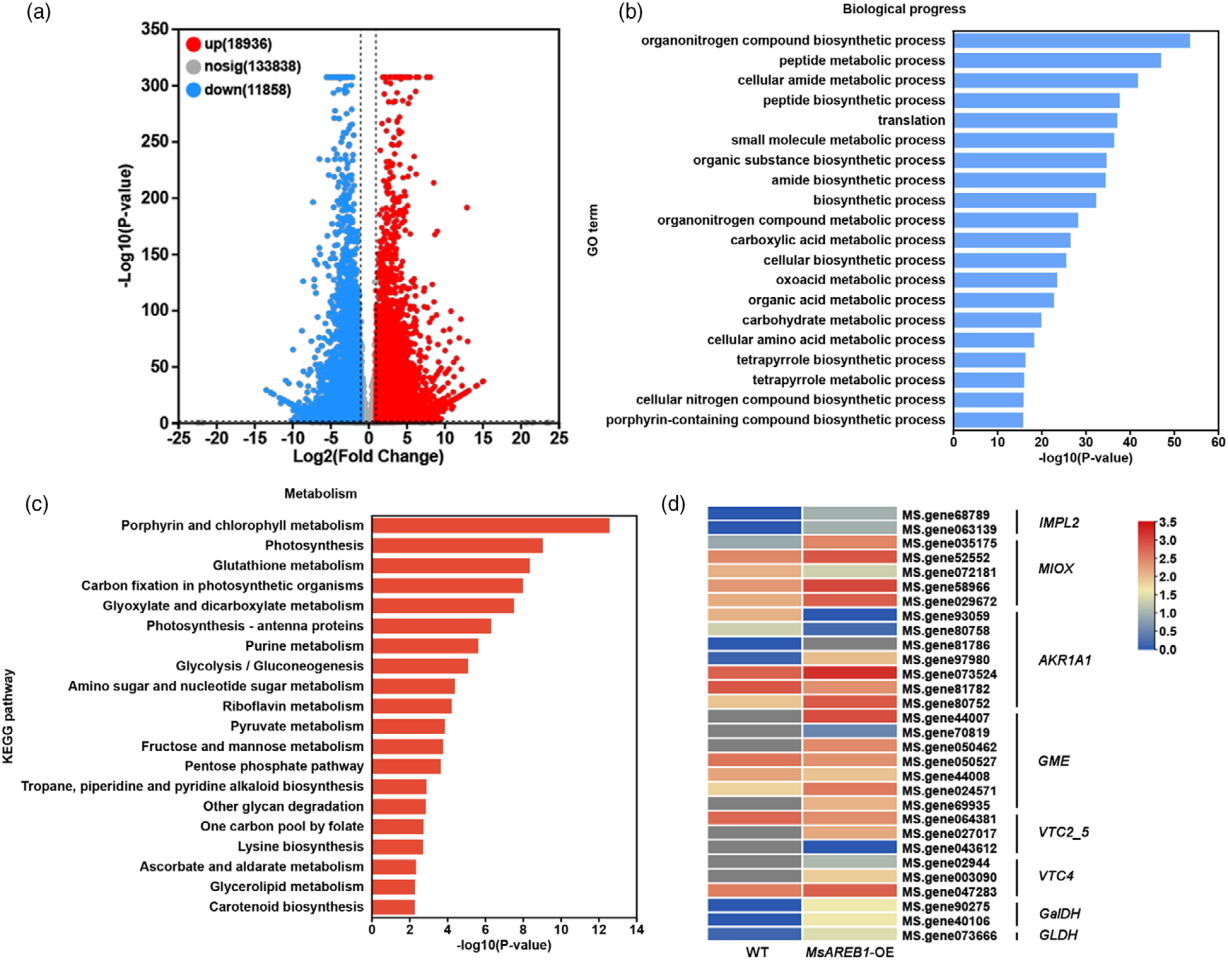
Overexpression of MsAREB1 induced the expression of genes involved in AsA biosynthesis in alfalfa. (a) Scatterplots comparing gene expression patterns in WT and MsAREB1‐OE line under normal conditions. Blue and red circles represent downregulated and upregulated genes. (b) GO analysis of the top 20 enrichments of upregulated DEGs in terms of biological process. (c) Top 20 enriched KEGG pathways among the upregulated DEGs in terms of metabolism. (d) Heat map of expression levels for genes related to AsA biosynthesis in WT and MsAREB1‐OE line. Genes are represented with their ID numbers and their annotations.

A total of 30 794 genes had altered transcription levels (fold change ≥2, corrected *P* ≤ 0.05) in *MsAREB1*‐OE alfalfa compared with the WT, with 18 936 genes upregulated and 11 858 genes downregulated (Figure 3a). In the first 20 enriched Gene Ontology (GO) analyses, differentially expressed genes (DEGs) were involved in various biological processes, with the ‘organonitrogen compound biosynthetic’ class having the highest proportion, followed by peptide biosynthetic, organic substance biosynthetic process, amide biosynthetic process, carboxylic acid metabolic, organic acid metabolic, metabolic carbohydrate and cellular amino acid metabolism (Figure 3b). These results show that MsAREB1 may significantly affect biosynthesis and metabolism. Furthermore, half of the top 20 enriched Kyoto Encyclopedia of Genes and Genomes (KEGG) pathways were associated with the metabolic processes of glutathione, amino sugars, nucleotide sugars, riboflavin, fructose, mannose, ascorbate and aldarate (Figure 3c). MsAREB1 may coordinate the response to CSA stress by regulating the levels of stress‐related substances. Under CSA stress, *MsAREB1* positively regulated AsA content in alfalfa (Figure 2i), and the AsA metabolic process was one of the most enriched pathways.

The transcriptome was screened for DEGs related to AsA biosynthesis, including *IMPL2*, *MIOX*, *AKR1A1*, *GME*, *VTC2_5*, *VTC4*, *GALDH* and *GDH* (Figure 3d). Among the family members of these genes, the gene with the highest expression level was selected for RT‐qPCR for further validation. Compared with the WT, the *IMPL2* (MS.gen58789.t1), *MIOX* (MS.gen58966.t1), *AKR1A1* (MS.gen073524.t1), *GME* (MS.gen44007.t1), *VTC2_5* (MS.gen027017.t1), *VTC4* (MS.gen047283.t1), *GALDH* (MS.gen90275.t1) and *GDH* (MS.gen073666.t1) expression levels in *MsAREB1*‐OE lines were significantly upregulated (Figure S4). Therefore, MsAREB1 may promote AsA accumulation by regulating the expression of these genes. In addition, the transcription levels of AsA biosynthesis‐related genes were analysed under CSA stress and ABA treatment. CSA stress induced *IMPL2*, *MIOX*, *AKR1A1* and *GME* transcription (Figure S5). ABA treatment also induced the *MIOX* transcription (Figure S6). *MIOX* (MS.gene58966.t1) was significantly upregulated under both CSA stress and ABA treatment. In a previous study, MS.gen58966.t1 was named *MsMIOX2* (Guo *et al*., 2024).

### MsAREB1 directly binds to the *MsMIOX2* promoter and activates its expression

**Figure 4:**
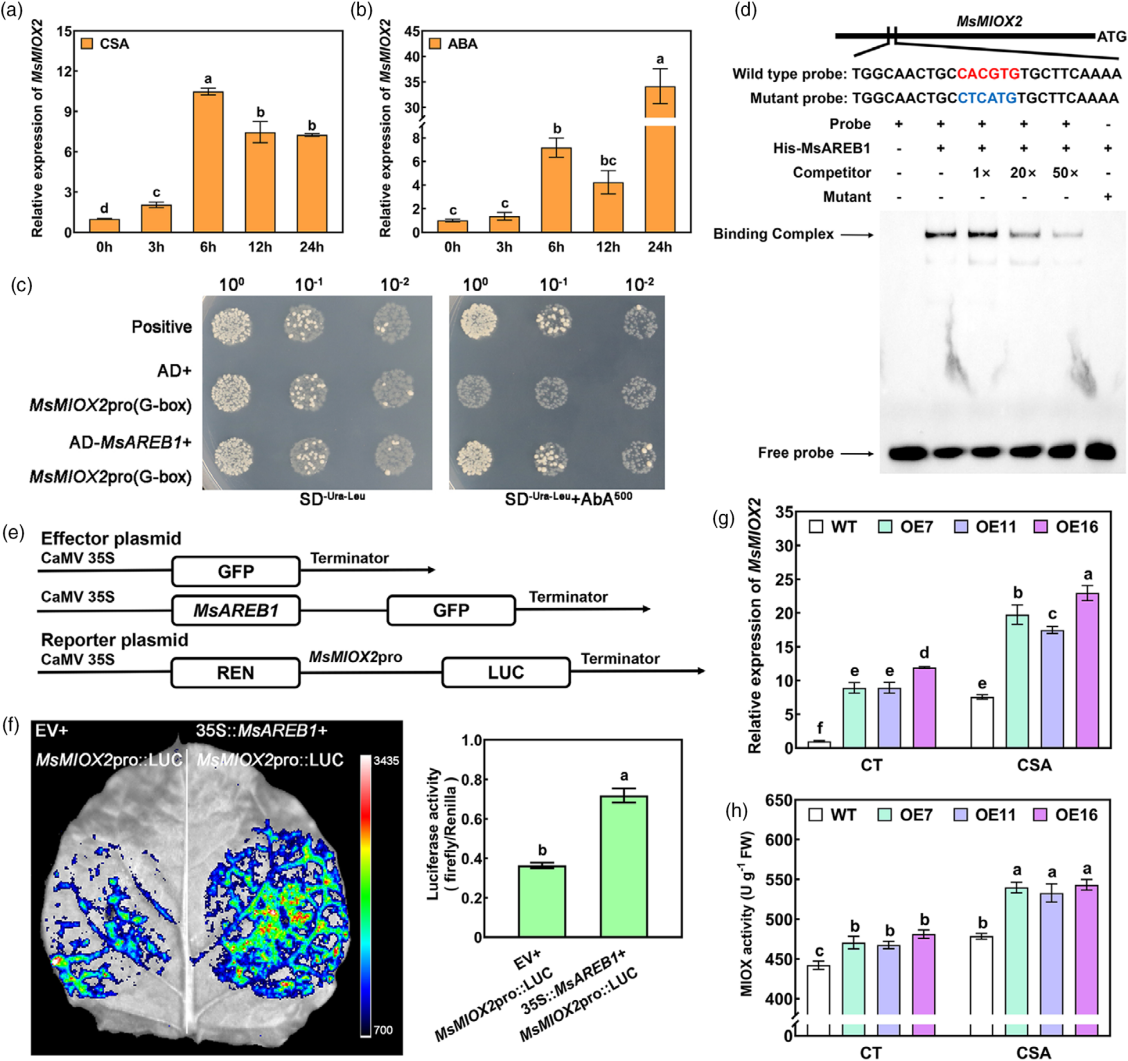
MsAREB1 binds to and activates the promoter of MsMIOX2. (a) MsMIOX2 expression in WT alfalfa under CSA stress. (b) MsMIOX2 expression in WT alfalfa under ABA treatment. (c) Yeast one‐hybrid (Y1H) assay to validate MsAREB1 binding to the G‐box element of MsMIOX2 promoter. (d) Electrophoretic mobility shift assay (EMSA) of the interaction between MsAREB1 and the G‐box (CACGTG) element of MsMIOX2 promoter. (e) Schematic diagrams of the effector (EV and 35S::MsAREB1) and reporter (MsMIOX2pro::LUC) plasmids used for dual‐luciferase reporter assay (dual‐LUC). REN, Renilla luciferase; LUC, firefly luciferase. (f) LUC assay to identify the activation of MsAREB1 on the promoter of MsMIOX2. (g, h) MsMIOX2 expression and MIOX activity of WT and MsAREB1‐OE alfalfa in the control and under CSA treatment. Data are mean ± SE (*n* = 3). Bars with different letters indicate a significant difference at *P* < 0.05.

Consistent with *MsAREB1*, *MsMIOX2* expression was significantly upregulated under CSA stress and ABA treatment (Figure 4a,b). The yeast one‐hybrid (Y1H) assay showed that, compared to the control group, yeast cells grew normally when the AD–MsAREB1 fusion protein was bound to the G‐box element in the *MsMIOX2* promoter (Figure 4c).

The electrophoretic mobility shift assay (EMSA) indicated that the His–MsAREB1 fusion protein bound to the G‐box element in the *MsMIOX2* promoter region. However, the addition of competitive probes decreased binding strength. After mutation of this element, no binding effect was observed (Figure 4d). Thus, MsAREB1 is directly bound to the *MsMIOX2* promoter. Additionally, a dual‐luciferase (LUC) assay was performed on tobacco leaves to verify whether MsAREB1 positively regulates the *MsMIOX2* promoter. Figure 4e shows the effectors and reporters. MsAREB1 strongly induced the expression of the *MsMIOX2* promoter. When 35S::*MsAREB1*–GFP and *MsMIOX2*pro::LUC were co‐expressed, the fluorescence effect on the leaves was more pronounced, and the ratio of LUC/REN was significantly higher than that of the control group (Figure 4f). These results indicate that MsAREB1 directly binds to the promoter region of *MsMIOX2*, thereby activating *MsMIOX2* transcription in vitro and in vivo.

To determine whether MsAREB1 promotes AsA accumulation by activating *MsMIOX2* transcription, *MsMIOX2* expression in WT and *MsAREB1*‐OE lines was investigated using RT‐qPCR, and the enzyme activity of MIOX encoded by *MsMIOX2* was determined. Under control conditions, *MsMIOX2* expression and MIOX enzyme activity in *MsAREB1*‐OE lines were significantly higher than those in the WT. After CSA stress, the difference became more significant (Figure 4g,h). These results show that MsAREB1 overexpression enhances MIOX activity by activating *MsMIOX2* transcription under CSA stress.

### *MsMIOX2* enhances tolerance to CSA stress by promoting AsA biosynthesis and inhibiting ROS production

**Figure 5:**
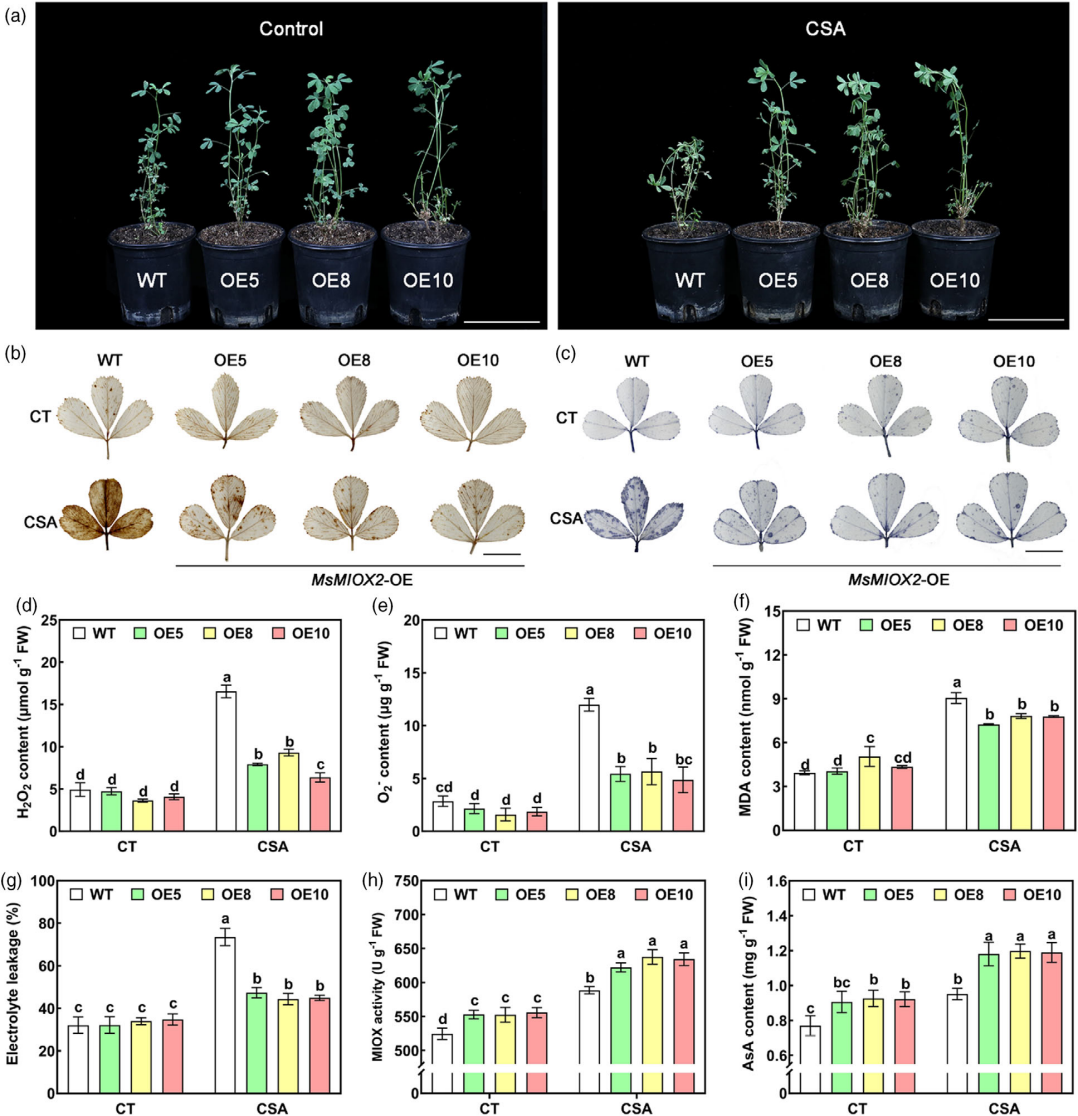
Overexpression of MsMIOX2 enhances CSA stress tolerance in alfalfa. (a) Phenotypes of WT and MsMIOX2‐OE alfalfa in Control and CSA treatment. Scale bars: 10 cm. (b, c) DAB and NBT staining for visualising the accumulation of ROS in Control and CSA treatment. Scale bars: 1 cm. (d–i) Determination of H_2_O_2_ content, O_2_^‐^ content, MDA content, electrolyte leakage, MIOX activity and AsA content in the control and under CSA treatment. Data are mean ± SE (*n* = 3). Bars with different letters indicate significant differences at *P* < 0.05.

Three *MsMIOX2*‐OE lines (OE5, OE8 and OE10) were used for further validation to determine the function of MsMIOX2 in CSA stress. Under CSA stress, the leaf tips were curved and wilted in the WT. In contrast, the growth of *MsMIOX2*‐OE transgenic alfalfa was significantly better than that of the WT (Figure 5a). The degree of DAB and NBT staining in the leaves of the *MsMIOX2*‐OE lines was weaker than that of the WT under CSA stress (Figure 5b,c), and the H_2_O_2_ and $O_{2}^{-}$ contents were significantly lower than those of the WT (Figure 5d,e). Similarly, the MDA content and electrolyte leakage were lower in *MsMIOX2*‐OE lines than in WT plants under CSA stress (Figure 5f,g). MIOX enzyme activity and AsA content were assayed to determine whether MsMIOX2 is a key enzyme in the MI pathway that promotes AsA accumulation. MIOX activity and the AsA content of *MsMIOX2*‐OE lines were significantly higher than those of the WT under control conditions. After CSA stress, the differences in MIOX activity and AsA content between *MsMIOX2*‐OE lines and the WT were more pronounced (Figure 5h,i).

### MsILR3 interacts with MsAREB1

MsAREB1 was used as bait to screen the alfalfa cDNA library using Y2H to further explore the regulatory mechanism of AsA biosynthesis in alfalfa. The bHLH protein (MS.gene031275.t1) was obtained, and domain analysis showed that MS.gene031275.t1 contained an HLH domain (75–126 amino acids, Figure S7a). Phylogenetic analysis showed that MS.gene031275.t1 had the highest homology with MtILR3 of *Medicago truncatula*; therefore, it was named *MsILR3* (Figure S7b,c). The ORF of *MsILR3* was 690 bp and encoded 229 amino acids.

**Figure 6:**
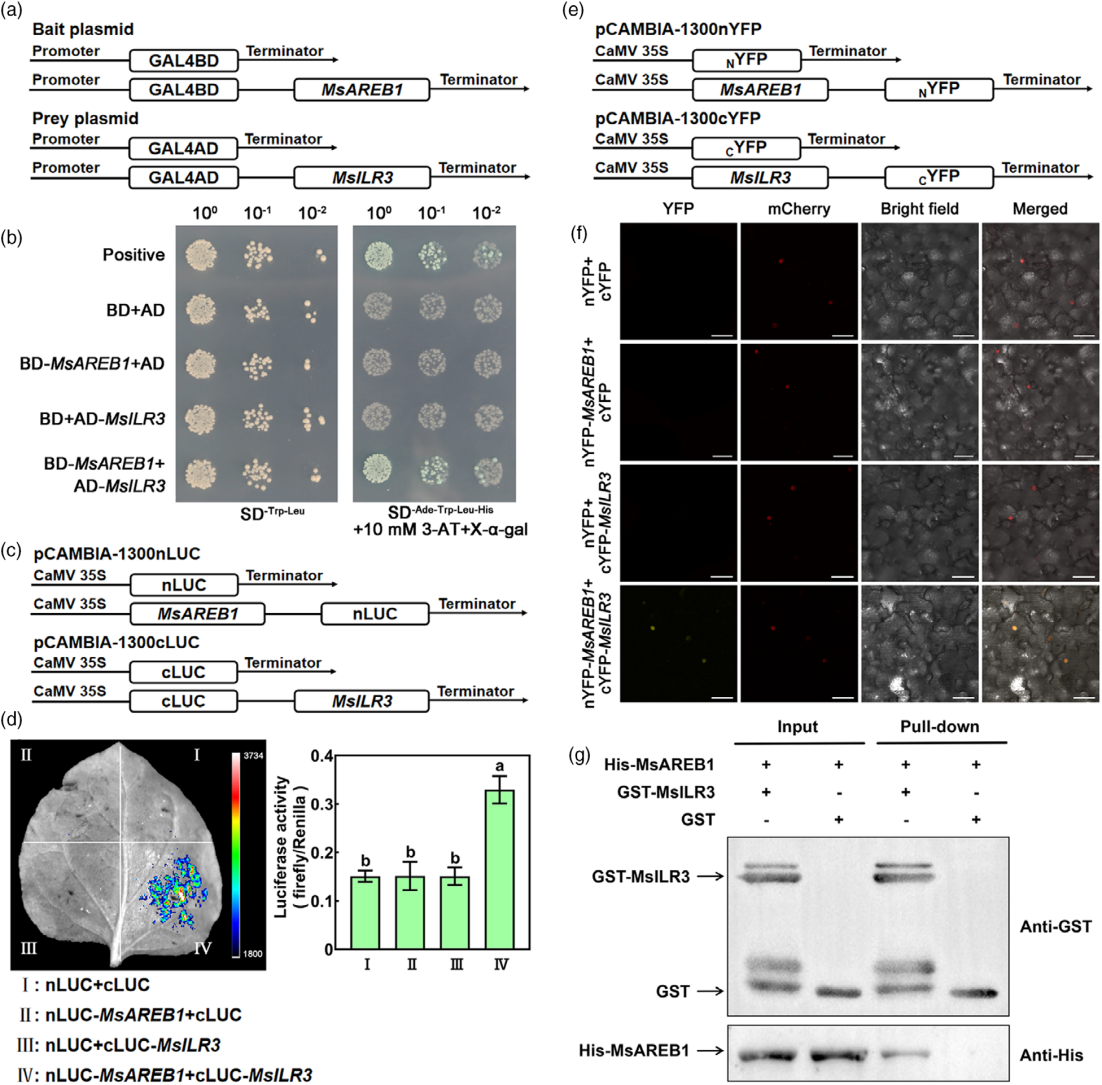
MsILR3 interacts with MsAREB1. (a) Conventional diagram of yeast two‐hybrid (Y2H) assay. BD‐MsAREB1 as the bait, AD‐MsILR3 as the prey. (b) Y2H assay indicates that MsILR3 interacts with MsAREB1 in yeast in vivo. (c) Schematic map of luciferase complementation imaging (LCI) assay. (d) LCI assay of MsILR3 interaction with MsAREB1 in Nicotiana benthamiana leaves. Data are mean ± SE (*n* = 3). Bars with different letters indicate a significant difference at *P* < 0.05. (e) Diagrammatic drawing of bimolecular fluorescence complementation (BiFC) assay. (f) BiFC assay infers the interaction between MsAREB1 and MsILR3 in Nicotiana benthamiana leaves. The empty vector (nYFP and cYFP) was used as the control. The mCherry protein indicates nucleus localisation. Scale bars: 50 μm. (g) In vitro pull‐down assay verifying the interaction between MsAREB1 and MsILR3.

The Y2H revealed that yeast transformed with AD–MsILR3 and BD–MsAREB1 grew on the quadruple dropout medium (SD/‐Ade/‐Trp/‐Leu/‐His) and exhibited blue staining with X‐ɑ‐gal (Figure 6a,b). The Y2H assay also showed that MsAREB1 interacts with MsILR3. Luciferase complementation imaging (LCI) and bimolecular fluorescence complementation (BiFC) assays confirmed the interaction between MsILR3 and MsAREB1 in tobacco. The LCI assay indicated that the LUC fluorescence signal was only detected in nLUC–MsAREB1 and cLUC–MsILR3 co‐transformed tobacco leaves and that the LUC/REN ratio was significantly different from that of the other groups (Figure 6c,d). Consistent with MsAREB1, MsILR3 was localised to the nucleus (Figure S8). The BiFC assay results indicated that the yellow fluorescence signal was localised exclusively to the nuclei of nYFP–MsAREB1 and cYFP–MsILR3 co‐transformed tobacco leaves, suggesting a physical interaction between the two within the nucleus (Figure 6e,f). Moreover, a pull‐down assay was performed, in which the GST–MsILR3 fusion protein was precipitated as a protein complex with His–MsAREB1 purified protein rather than His protein alone using GST resin. MsILR3 physically interacted with MsAREB1 in vitro (Figure 6g). In summary, MsAREB1 and MsILR3 interact both in vivo and in vitro.

### MsILR3 inhibits and competes with MsAREB1 for binding to the *MsMIOX2* promoter

**Figure 7:**
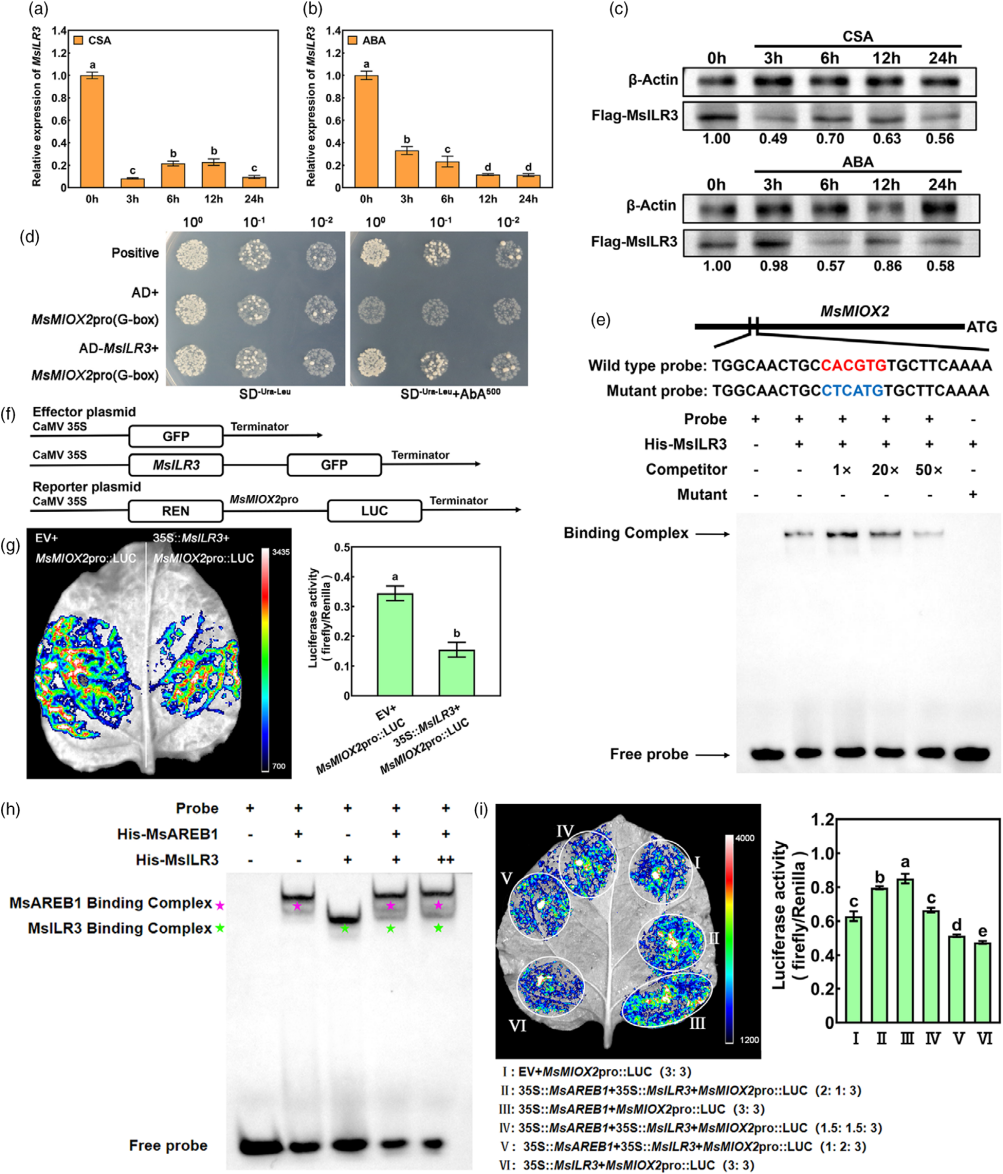
MsILR3 inhibits and competes with MsAREB1 for binding to the MsMIOX2 promoter. (a) MsILR3 expression in alfalfa under CSA stress. (b) MsILR3 expression in alfalfa under ABA treatment. (c) MsILR3 protein abundance under CSA stress and ABA treatment. (d) Y1H assay to validate MsILR3 binding to the G‐box element of MsMIOX2 promoter. (e) EMSA assay of the interaction between MsILR3 and the G‐box (CACGTG) element of MsMIOX2 promoter. (f) Schematic diagrams of the effector (EV and 35S::MsILR3) and reporter (MsMIOX2pro::LUC) plasmids used for dual‐LUC assay. REN, Renilla luciferase; LUC, firefly luciferase. (g) LUC assay to identify the inhibition of MsILR3 on the promoter of MsMIOX2. (h) EMSA assays show the competitive binding between His‐MsILR3 and His‐MsAREB1 to the G‐box element of the MsMIOX2 promoter. Purple and green asterisks indicate the positions of His‐MsAREB1 and His‐MsILR3 proteins, respectively. The concentration of His‐MsILR3 protein used in the competitive assay was 1‐ and 2‐fold MsAREB1. (i) LUC assay showing that the interaction of MsILR3 with MsAREB1 hinders the activation effects of MsAREB1 on the transcription of MsMIOX2. Data are mean ± SE (*n* = 3). Bars with different letters indicate a significant difference at *P* < 0.05.

In contrast to *MsMIOX2*, *MsILR3* expression was significantly downregulated by CSA stress and ABA treatment (Figure 7a,b). Immunoblot analysis showed that MsILR3 protein abundance gradually decreased under CSA stress and ABA treatment in MsILR3‐Flag transgenic plants (Figure 7c). bHLH TFs can affect the expression of downstream genes by binding to G‐box elements (Hao *et al*., 2021). Therefore, MsILR3 and *MsMIOX2* promoters possibly interact directly, and MsILR3 could negatively regulate *MsMIOX2* expression. The yeast cells grew normally in the Y1H assay when the AD–MsILR3 fusion protein was bound to the G‐box element in the *MsMIOX2* promoter region compared to the control group (Figure 7d). The EMSA assay showed that the His–MsILR3 fusion protein bound to the G‐box element in the *MsMIOX2* promoter region. However, the binding effect decreased as the concentration of the competitive probes increased. No binding effect was observed after the mutation of the element (Figure 7e). Thus, MsILR3 is bound directly to the G‐box element in the *MsMIOX2* promoter region. A LUC assay was performed to validate the mechanism by which MsILR3 regulates *MsMIOX2* expression. The effectors and reporters are shown in Figure 7f. When 35S::*MsILR3*–GFP and *MsMIOX2*pro::LUC were co‐expressed, the fluorescence effect of the leaves was weaker than that of the control group, and the LUC/REN ratio was significantly lower than that of the control group (Figure 7g), indicating that MsILR3 inhibited *MsMIOX2* expression. These results demonstrate that MsILR3 directly binds to the *MsMIOX2* promoter and inhibits its transcription.

Moreover, owing to the interaction between MsILR3 and MsAREB1, both MsAREB1 and MsILR3 could directly bind to the G‐box element to regulate *MsMIOX2* expression. We hypothesized that they might compete for binding. To verify this, we performed EMSA assays and observed that when MsAREB1 and MsILR3 were simultaneously bound to the probes, the binding signal of both MsAREB1 and MsILR3 became weaker, but overall, the binding ability of MsAREB1 was superior to MsILR3. Moreover, as the amounts of MsILR3 increased, the binding signal became deeper (Figure 7h). The results indicated that the interaction between MsAREB1 and MsILR3 affected their binding to the MsMIOX2 promoter. To determine whether ABA affected the interaction of MsAREB1 and MsILR3, a LCI assay was used (Figure S9). The fluorescence intensity of activated nLUC–MsAREB1 and cLUC–MsILR3 in the LCI assay was not notably changed in response to ABA. The transcript and protein levels of MsAREB1 were significantly elevated under CSA stress, while MsILR3 showed the contrary trend (Figures 1e–g and 7a–c). To examine the physical interaction between MsAREB1 and MsILR3 under stress, we observed the subcellular localisation of MsAREB1 and MsILR3 under CSA stress and found that both were localized in the nucleus, which was consistent with normal conditions (Figure S10). Meanwhile, the BiFC assay revealed that MsAREB1 and MsILR3 interacted in the nucleus under CSA stress (Figure S11). Therefore, CSA stress did not affect their interactions. Subsequently, 35S::MsAREB1–GFP and 35S::MsILR3–GFP were co‐transformed with *MsMIOX2*pro::LUC into tobacco leaves. When MsAREB1 was combined with MsILR3, the enhancement effect of MsAREB1 was weakened, the inhibitory effect of MsILR3 was alleviated and the LUC fluorescence was restored to the control level (Figure 7i). These results indicate that antagonistic interactions between MsILR3 and MsAREB1 affect the regulation of the *MsMIOX2* promoter.

## Discussion

Mining and identifying the key genes of alfalfa that respond to CSA stress is conducive to the genetic improvement of alfalfa and has great significance for the effective use of saline–alkali soil in cold regions for agricultural production. CSA stress can trigger excessive ROS production, stimulate membrane lipid peroxidation and induce osmotic stress in plant cells, thus affecting plant growth and development and even leading to death (Geng *et al*., 2021; Rao *et al*., 2023). The antioxidant capacity of plants is positively correlated with their ability to resist stress. AsA is a multidimensional plant antioxidant that scavenges ROS, acts as a cofactor for various enzymes, regulates gene expression and plays an active role under abiotic stress (Singh *et al*., 2024). In the present study, AsA accumulated significantly under CSA stress, indicating that variation in AsA content may be a primary factor in alfalfa resistance to CSA stress.

The MI pathway, which is involved in AsA biosynthesis, has been extensively studied (Lorence *et al*., 2004). Lorence *et al*. (2004) first suggested a role for MI in AsA biosynthesis in 2004, when a 2‐ to 3‐fold increase in AsA levels was found in *Arabidopsis* homozygous lines overexpressing *MIOX4*. Subsequently, Endres and Tenhaken (2009) proposed that overexpression of the MIOX gene in *Arabidopsis* could not increase the content of AsA. However, Lisko *et al*. (2013) demonstrated later that *Arabidopsis* overexpressing *AtMIOX4* contained higher levels of AsA content, exhibited growth and biomass accumulation in aerial and root tissues and had greater tolerance to abiotic stress. Likewise, overexpression of *SlMIOX4* in tomato significantly increased AsA content in its leaves and fruits (Munir *et al*., 2020). In this study, overexpression of *MsMIOX2* in alfalfa significantly increased MIOX activity and AsA content. Moreover, *MsMIOX2* overexpression in alfalfa enhanced its ability to scavenge ROS under CSA stress, weakened membrane lipid peroxidation and enhanced CSA stress resistance. Overall, the results revealed that *MsMIOX2* overexpression enhanced the synthesis of AsA and increased the resistance to CSA stress in alfalfa.

ABA plays a crucial role in the signalling pathway in response to stress (Parwez *et al*., 2022). Changes in ABA content under abiotic stress can alter the AsA content. Drought stress increases AsA accumulation, enhancing AsA biosynthesis and drought resistance in *Arabidopsis* and maize by regulating *PTPN* expression (Zhang *et al*., 2020). Furthermore, ABA‐induced AsA accumulation in tomatoes is mediated by the *SlMAPK8*–*SlARF4*–*SlMYB11* module under drought stress (Xu *et al*., 2022). Additionally, Yu *et al*. (2024) demonstrated the central role of the *BcMYB30*–*BcSRC2*–*BcAPX4* regulatory module in increasing AsA content in pak choi to respond to ABA‐mediated drought stress (Yu *et al*., 2024). However, in kiwifruit, ABA reduces AsA content by inhibiting *AceMYBS1* expression (Liu *et al*., 2022). In the present study, ABA content increased significantly under CSA stress, consistent with the change in AsA content. Interestingly, MsAREB1, an AREB/ABF TF, was discovered, which was significantly induced by CSA stress and ABA treatment. Compared with the WT, *MsAREB1‐*OE transgenic alfalfa had higher AsA content and a stronger ability to resist CSA stress. These results suggest that MsAREB1 plays a pivotal role in ABA‐mediated AsA biosynthesis under CSA stress. Additionally, *MsMIOX2* was significantly upregulated in *MsAREB1*‐OE lines compared to the WT. The expression pattern of *MsMIOX2* was consistent with that of *MsAREB1*, which could be induced by CSA stress and ABA treatment. Additionally, MsAREB1 directly binds to the promoter region of *MsMIOX2* and positively regulates its expression. In line with this, *MsMIOX2* expression and MIOX activity in *MsAREB1*‐OE transgenic alfalfa were significantly higher than those in the WT, consistent with the changes in AsA content. These results indicate that MsAREB1‐induced AsA accumulation under CSA stress was partly due to the MI pathway activation, which was caused by positively regulating *MsMIOX2* expression.

MsILR3 is one of the most widely distributed bHLH TFs in plants. ILR3 interacts with other TFs in *Arabidopsis* and is involved in different metabolic pathways, including iron and ROS homeostasis, auxin responsiveness and stress responses (Akmakjian *et al*., 2021; Gao *et al*., 2020; Tissot *et al*., 2019). Additionally, an *RhILR3*–*RhLOL1* regulatory module mediates cytokinin‐induced petal drops in roses by regulating the expression of Aux/IAA genes (Jiang *et al*., 2022). Recently, it was reported that MdILR3L can form a complex with MdCPCL, significantly enhancing the transcription of the downstream target gene *MdGLDH* and facilitating the synthesis of AsA in apple (Zou *et al*., 2024). However, the involvement of MsILR3 in plant responses to abiotic stress remained unknown. This study identified the transcription factor MsILR3. Using Y2H, LCI, BiFC and pull‐down, we confirmed that MsILR3 interacted with MsAREB1 both in vitro and in vivo (Figure 6). In addition, *MsILR3* expression significantly decreased under CSA stress and ABA treatment, in contrast to the expression trends of *MsAREB1*. EMSA assays demonstrated that MsILR3 binds the MsMIOX2 promoter by directly competing with MsAREB1 (Figure 7h). Further, LUC assays confirmed that the presence of MsILR3 hindered the activating effect of MsAREB1 on MsMIOX2 transcription (Figure 7i). This mechanism of competitive promoter occupancy suggested that plants regulate their stress response mechanisms by balancing the transcriptional activities of MsAREB1 and MsILR3 according to environmental needs.

**Figure 8:**
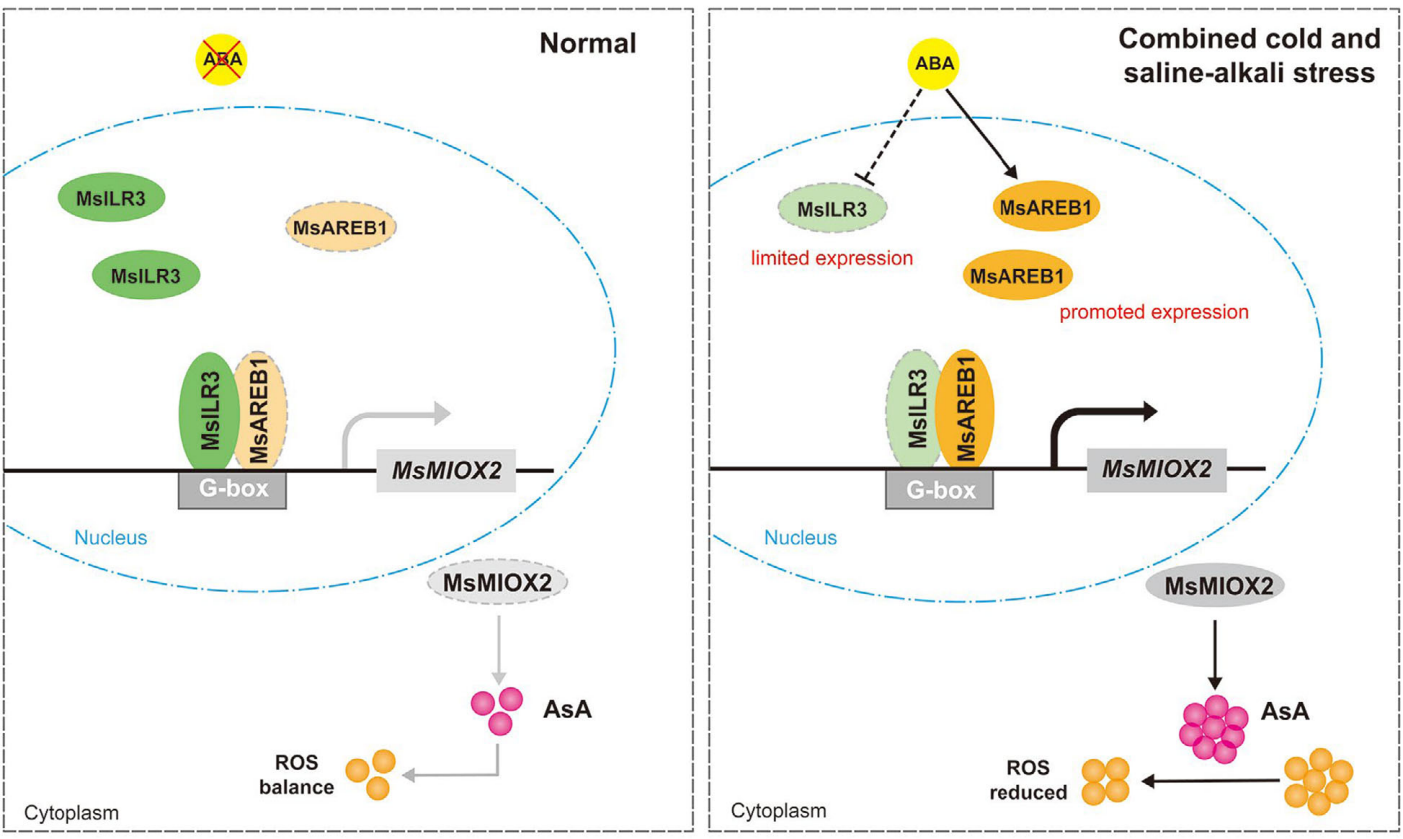
Model of the regulation mechanism of the MsAREB1‐MsILR3 module in resistance to CSA stress in alfalfa by modulating the expression of MsMIOX2. Under normal conditions, the expression of MsAREB1 and MsILR3 reaches a balanced state, and the antagonism between them makes AsA synthesis reach a relative equilibrium. However, CSA stress activates the ABA signalling pathway, which induces and inhibits the expression of MsAREB1 and MsILR3, respectively. Meanwhile, MsAREB1 protein interacts with MsILR3 protein to form the MsAREB1‐MsILR3 protein complex, which promotes MsAREB1 and impedes MsILR3 binding to the promoter of MsMIOX2, respectively. This eventually leads to increased AsA content, ROS elimination ability and resistance to CSA stress.

MIOX is a key enzyme in the MI pathway for AsA biosynthesis and may be one of the primary mechanisms by which *MsAREB1* regulates the increase in AsA content under CSA stress. Based on the study results, a model was proposed to elucidate the regulatory mechanism of *MsAREB1*–*MsMIOX2* in regulating AsA synthesis in alfalfa in response to CSA stress (Figure 8). Under normal conditions, only a small amount of MsAREB1 was able to bind to the promoter of *MsMIOX2*, MsILR3 was able to form a complex with MsAREB1 and impede the activation effect of MsAREB1, and the rest of MsILR3 was able to compete for the G‐box site of the promoter of *MsMIOX2* by competing with MsAREB1 and repressing the transcription of *MsMIOX2*. Under CSA stress, ABA signalling was activated and MsAREB1 expression was significantly up‐regulated. The high‐level expression of MsAREB1 protein activated the transcription of *MsMIOX2*. In contrast, MsILR3 was significantly down‐regulated under CSA stress. In this case, almost all MsILR3 proteins were to interact with MsAREB1 to form a complex, and even fewer MsILR3 proteins competed with MsAREB1 to bind to the *MsMIOX2* promoter, leading to more reduced inhibitory effects on the transcription of *MsMIOX2*, which ultimately resulted in the alfalfa exhibiting high levels of AsA content and ROS scavenging ability under CSA stress.

In conclusion, the results of this study reveal the complex signalling pathway of AsA synthesis in alfalfa in response to CSA stress and provide a promising avenue for the antagonistic regulation of AsA biosynthesis through the MsAREB1–MsILR3 module. Furthermore, this study lays a theoretical foundation for the genetic improvement of alfalfa stress‐tolerant varieties and provides a scientific foundation for increasing the yield of forage in saline–alkali soils in cold regions. This study primarily focussed on the MI pathway. Therefore, whether regulatory modules exist that jointly control AsA biosynthesis through other pathways remains a topic for further exploration.

## Materials and methods

### Plant materials and growth conditions

Zhaodong (a resistant alfalfa variety) was selected to undergo CSA stress. The culture method and stress conditions have been described previously (Liu *et al*., 2024). Alfalfa seeds were germinated in pots filled with nutrient soil and cultured under long‐day photoperiod conditions (25 °C, 16 h light/20 °C, 8 h dark cycle, 225 μmol m^−2^ s^−1^). After 50 days of culture, the alfalfa seedlings were transferred to the incubator at 0 °C for cold stress and irrigated with 200 mm of saline–alkali mixture (NaHCO_3_: Na_2_CO_3_: NaCl: Na_2_SO_4_ at a molar ratio of 2:1:2:1, Na^+^ concentration, 200 mm) for saline–alkali stress. The roots, stems, leaves and flowers of the alfalfa were sampled. Alfalfa was subjected to CSA stress for 0, 3, 6, 12 and 24 h. For ABA treatment, alfalfa was treated with 100 μM ABA solution and sampled at 0, 3, 6, 12 and 24 h.

### RNA extraction and RT‐qPCR

Total RNA was extracted using the RNAprep Pure Plant Kit (DP432; TIANGEN, Beijing, China) according to the manufacturer's instructions. cDNA was acquired by reverse transcription using *EasyScript*® One‐Step gDNA Removal and cDNA Synthesis SuperMix (AE311; TransGen Biotech, Beijing, China). RT‐qPCR assays were performed as previously described (Guo *et al*., 2023). Primers used for this assay are listed in Supplemental Table S1. At least three biological replicates were used for each experiment.

### Gene cloning and sequence analysis

The full‐length coding sequences (CDS) of *MsAREB1* and *MsILR3* were amplified from alfalfa cDNA using PCR. The SMART website was used to analyse the structural domains of MsAREB1 and MsILR3 (http://smart.embl‐heidelberg.de/). Phylogenetic trees for MsAREB1 and MsILR3 were constructed using MEGA11.0.13 software.

### Subcellular localisation and transcriptional activity

The *MsAREB1* and *MsILR3* CDS were integrated into a GFP vector. The recombinant plasmid was co‐introduced with the nuclear marker (AtH2B–mcherry) into the tobacco leaves. Laser confocal microscopy (Leica Microsystems, Wetzlar, Germany) was used to detect fluorescence signals.

Full‐length N‐ (1–1041 bp) and C‐terminals (1042–1305 bp) of *MsAREB1* were integrated into the pGBKT7 (BD) vector. The transcriptional activities of MsAREB1 were characterised by transfecting BD–MsAREB1 (FL), BD–MsAREB1 (C), BD–MsAREB1 (N), and BD into Y2HGold yeast. Transcriptional activity was characterised based on the growth of SD/−Trp and SD/−Trp/−His (X‐ɑ‐gal) in the medium at 30 °C for 3 days.

### ABA and AsA content measurement

The ABA content was measured using a commercial kit (YJ077235, Yuanju Biotech, Shanghai, China) according to the manufacturer's instructions. AsA was detected using high‐performance liquid chromatography as previously described (Liu *et al*., 2015). A 10 μL sample filtered through a 0.45 μM filter was injected onto an XDB‐C18 column (4.6 mm × 250 mm, SHIMADZU, Kyoto, Japan) and detected using a high‐performance liquid chromatograph (LC‐2050, SHIMADZU, Kyoto, Japan). The column was run at 25 °C at a flow rate of 0.7 mL min^−1^ with 20 mm of sodium dihydrogen phosphate and methanol at a volume ratio of 98:2 as the mobile phase. The AsA content was calculated based on the OD value at 245 nm. Data were recorded and processed using the LC‐2050‐System software.

### Construction and phenotypic analysis of transgenic alfalfa

The CDS of *MsAREB1* was inserted into the pCAMBIA1302 vector driven by the CaMV35S promoter. The *MsAREB1* overexpression transgenic alfalfa T_0_ generation was obtained from alfalfa leaves using *Agrobacterium*‐mediated transformation. Three *MsMIOX2*‐OE lines (OE5, OE8, and OE10) were constructed based on a previous study (Guo *et al*., 2024). Transgenic alfalfa overexpressing *MsAREB1* and *MsMIOX2*, as well as WT plants, were cut for phenotypic analysis of CSA stress resistance. The cutting and cultivation of transgenic alfalfa and WT plants were performed as previously described (Guo *et al*., 2024). Four weeks after transplantation, plants of similar size were selected from each line for CSA stress. Phenotypic and physiological assay materials of transgenic alfalfa and WT plants under CSA stress were obtained as previously described (Liu *et al*., 2024). The treatment conditions for CSA stress were as previously described in the ‘Plant materials and growth conditions’ section. The transgenic alfalfa before and after CSA stress for 5 days was phenotyped, and the leaves before and after stress were collected for physiological assays.

### Immunoblot analysis

The CDS of MsAREB1 was inserted into a GFP vector containing a GFP tag. The CDS of MsILR3 was inserted into the pEarlyGate100 vector containing a Flag tag. The recombinant plasmid was introduced into the tobacco leaves. Plant proteins were extracted using protein lysate (50 mm Tris–HCl, 150 mm NaCl, 1 mm EDTA, 1% Triton X‐100, and 1% PMSF). The tobacco infected with MsAREB1‐GFP and MsILR3‐Flag *Agrobacterium* was subjected to CSA stress and ABA treatment at 0, 3, 6, 12, and 24 h. MsAREB1 and MsILR3 proteins were detected with anti‐GFP and anti‐FLAG antibodies. MsAREB1 and MsILR3 protein levels were normalized to β‐Actin. Protein levels at 0 h were set to 1.0. The immunoblotting results were quantitatively analysed using ImageJ software.

### Physiological determination and histochemical staining

ROS, especially H_2_O_2_ and $O_{2}^{-}$, MDA, and electrolyte leakage are essential indicators of stress damage. Histochemical staining with DAB and NBT was performed to detect H_2_O_2_ and $O_{2}^{-}$, respectively (Sun *et al*., 2017). The MDA, H_2_O_2_, and O_2_^−^ contents were determined using a microfabrication kit (Sangon Biotech, Shanghai, China). Electrolyte leakage was determined as previously described (DaCosta *et al*., 2004). MIOX activity was determined using a Plant MIOX ELISA kit (MEIMIAN, Jiangsu, China).

### RNA sequencing and analysis

RNA sequencing was performed on *MsAREB1*‐OE alfalfa and WT plants under normal growth conditions to explore the molecular mechanism of MsAREB1 in transcriptional regulation and identify potential target genes regulated by MsAREB1. Four‐week‐old leaves of WT and *MsAREB1*‐OE41 transgenic alfalfa were collected for RNA sequencing analysis. Three independent biological replicates were used for each genotype. The RNA sequencing results were analysed as previously described (Liu *et al*., 2024). The sequence data were uploaded to GenBank of NCBI (https://www.ncbi.nlm.nih.gov/) under accession no. PRJNA1242565. Heat maps were drawn using TBtools.

### Y1H assay

The *MsMIOX2* promoter contains a G‐box element, which is a potential binding site for MsAREB1 (Guo *et al*., 2024). Therefore, a yeast one‐hybrid (Y1H) assay was performed to further explore the regulatory relationship between MsAREB1 and the *MsMIOX2* promoter. Synthesised sequences of three tandem‐repeated G‐box elements (CACGTG) were inserted into the pAbAi vector as bait. The CDS of the TFs *MsAREB1* and *MsILR3* were inserted into the pGADT7 (AD) vector by homologous recombination to construct the prey. Co‐transformation of the bait and prey into Y1HGold yeast was performed using a Classic Yeast Transformation Kit (SK2400; Coolaber, Beijing, China). Recombinant yeast was cultured at 30 °C for 3 days in SD/‐Ura/‐Leu medium with or without AbA.

### Dual‐luciferase reporter assay

35S::*MsAREB1*–GFP, and 35S::*MsILR3*–GFP were constructed as effectors using 35S::GFP as a control. The recombinant vector pGreenII0800–LUC ligated to the *MsMIOX2* promoter served as a reporter. The effector and reporter were cotransfected into *Agrobacterium* GV3101(pSoup–p19) and transiently transformed into tobacco leaves. LUC imaging was performed using an imager (Tanon‐5200; Shanghai, China). Transient expression was assessed by measuring firefly and Renilla luciferase activities using a dual‐luciferase reporter gene assay kit (11402ES560; YEASEN, Shanghai, China).

### EMSA

The CDS of *MsAREB1* and *MsILR3* were inserted into the pCold–His vector to obtain the fusion plasmids, pCold–*MsAREB1* and pCold–*MsILR3*, which were transformed into Rosetta (DE3) cells. Recombinant His–MsAREB1 and His–MsILR3 protein expressions were induced at 16 °C for 12 h using 0.25 mm isopropyl β‐d‐1‐thiogalactopyranoside and purified using proteiniso® Ni‐NTA Resin (DP101; TransGen Biotech, Beijing, China). Probes containing G‐box and mutant elements were synthesised using biotin labelling (Sangon Biotech, Shanghai, China). Unlabelled probes were used as competitors. A chemiluminescent EMSA kit (GS009; Beyotime, Shanghai, China) was used for the EMSA.

### Y2H assay

A Y2H assay was performed to test the validity of the yeast library screening results and to elucidate the potential interaction between MsILR3 and MsAREB1. The Y2H screening assay was performed as previously described (Wang *et al*., 2023). The CDS of *MsILR3* and *MsAREB1* was constructed into AD and BD vectors, respectively, using homologous recombination. The AD–*MsILR3* and BD–*MsAREB1* plasmids were transformed into the yeast strain Y2HGold. Yeast cells were cultured on SD/‐Trp/‐Leu and SD/‐Ade/‐Trp/‐Leu/‐His (3‐AT+X‐ɑ‐gal) medium, respectively.

### LCI assay

The CDS of *MsAREB1* and *MsILR3* were inserted into pCAMBIA1300–nLUC and pCAMBIA1300–cLUC vectors, respectively. nLUC–*MsAREB1* and cLUC–*MsILR3* recombinant plasmids were transformed into *Agrobacterium* GV3101(psoup–p19) cells. Different combinations of the same volume were mixed to inject *Nicotiana benthamiana* leaves. nLUC–*MsAREB1* + cLUC, nLUC + cLUC–*MsILR3*, and nLUC + cLUC co‐infiltrated leaves were used as negative controls. LUC image observation and enzyme activity analysis were performed as described in the ‘Dual‐luciferase reporter assay’ section.

### BIFC assay

The CDS of *MsAREB1* and *MsILR3* were inserted into pCAMBIA1300–nYFP and pCAMBIA1300–cYFP vectors, respectively. nYFP–*MsAREB1* and cYFP–*MsILR3* recombinant plasmids were transformed into *Agrobacterium* GV3101(psoup–p19) cells. Different combinations of the same volume were mixed to inject *N. benthamiana* leaves. AtH2B–mcherry was co‐injected with each combination as a nuclear localisation marker. nYFP–*MsAREB1* + cYFP, nYFP + cYFP–*MsILR3*, and nYFP + cYFP‐co‐infiltrated leaves were used as negative controls. Laser confocal microscopy (Leica Microsystems) was used to detect fluorescence signals.

### Pull‐down assay

The GST fusion protein was used to analyse protein interactions. In the GST pull‐down assay, the full‐length CDS of *MsILR3* was inserted into the pGEX‐4 T‐1 vector, and the full‐length CDS of *MsAREB1* was inserted into the pCold vector. Recombinant proteins were used for the GST pull‐down assay (Oh *et al*., 2012). The interaction between MSILR3 and MsAREB1 was analysed using sodium dodecyl sulfate polyacrylamide gel electrophoresis and western blotting.

### Statistical analysis

All experiments were performed in at least three biological replicates using independent samples. Data are presented as the mean ± standard error of the three biological replicates. Bars with different letters indicate significant differences at *P* < 0.05. Statistical analysis was performed using a one‐way analysis of variance with Tukey's multiple comparison test.

## Conflict of interest

The authors declare no conflict of interest.

## Funding

This study was funded by the National Natural Science Foundation of China (NSFC) (U21A20182), the National Key R&D Program of China (2022YFE0203300) and the Scientific Research Foundation for Doctors of Harbin Normal University (XKB202214).

## Author contributions

W.G. and C.G. designed the study. W.G., Y.S. and J.C. performed the experiments. W.G. and Y.S. analysed the data. W.G. and C.G. wrote the manuscript. L.L., J.L., Y.R. and C.G. revised the manuscript. All authors have read and approved the final draft of the manuscript.

## Supporting information


**Figure S1** Heat map of AREB TFs derived from transcriptome data analysis.
**Figure S2** Conserved domain and sequence analyses of MsAREB1.
**Figure S3** The DNA identification of *MsAREB1* gene in *MsAREB1*‐OE transgenic alfalfa.
**Figure S4** The expression of key genes involved in AsA biosynthesis in WT and *MsAREB1*‐OE transgenic alfalfa.
**Figure S5** The expression of key genes involved in AsA biosynthesis in CSA stress‐treated alfalfa.
**Figure S6** The expression of key genes involved in AsA biosynthesis in ABA solution‐treated alfalfa.
**Figure S7** Conserved domain, phylogenetic and sequence analyses of MsILR3.
**Figure S8** LCI assay using nLUC‐MsAREB1 and cLUC‐MsILR3 constructs; *Agrobacterium* cultures were combined at a 1:1 (v/v) ± 2 μm ABA, then infiltrated into *Nicotiana benthamiana* leaves.
**Figure S9** Subcellular localisation of the 35S::*MsILR3*‐GFP fusion protein in *Nicotiana benthamiana* leaf epidermal cells.
**Figure S10** Subcellular localisation of the 35S::*MsAREB1*‐GFP and 35S::*MsILR3*‐GFP fusion protein in *Nicotiana benthamiana* leaf epidermal cells under CSA stress.
**Figure S11** BiFC assay infers the interaction between MsAREB1 and MsILR3 in *Nicotiana benthamiana* leaves under CSA stress.
**Table S1** Primers used in this study.

## Data Availability

The data that support the findings of this study are openly available in NCBI at http://www.ncbi.nlm.nih.gov/bioproject, reference number PRJNA1242565.
