# Supplementary material for: MsAREB1 enhances combined cold and saline–alkali stress tolerance by promoting ascorbic acid biosynthesis in alfalfa
Source: Plant Biotechnol J. 2025 May 26;23(8):3349–62. doi: 10.1111/pbi.70156 (PMC12310818; doi:10.1111/pbi.70156)
Supplement: Supplementary file 1 — Figure S1 Heat map of AREB TFs derived from transcriptome data analysis. Figure S2 Conserved domain and sequence analyses of MsAREB1. Figure S3 The DNA identification of MsAREB1 gene in MsAREB1‐OE transgenic alfalfa. Figure S4 The expression of key genes involved in AsA biosynthesis in WT and MsAREB1‐OE transgenic alfalfa. Figure S5 The expression of key genes involved in AsA biosynthesis in CSA stress‐treated alfalfa. Figure S6 The expression of key genes involved in AsA biosynthesis in ABA solution‐treated alfalfa. Figure S7 Conserved domain, phylogenetic and sequence analyses of MsILR3. Figure S8 LCI assay using nLUC‐MsAREB1 and cLUC‐MsILR3 constructs; Agrobacterium cultures were combined at a 1:1 (v/v) ± 2 μm ABA, then infiltrated into Nicotiana benthamiana leaves. Figure S9 Subcellular localisation of the 35S::MsILR3‐GFP fusion protein in Nicotiana benthamiana leaf epidermal cells. Figure S10 Subcellular localisation of the 35S::MsAREB1‐GFP and 35S::MsILR3‐GFP fusion protein in Nicotiana benthamiana leaf epidermal cells under CSA stress. Figure S11 BiFC assay infers the interaction between MsAREB1 and MsILR3 in Nicotiana benthamiana leaves under CSA stress. Table S1 Primers used in this study. [file PBI-23-3349-s001.doc]

**Supporting Information**

**Article title:**

**MsAREB1 enhances combined cold and saline–alkali stress tolerance by promoting ascorbic acid biosynthesis in alfalfa**

**Authors:**

**Weileng Guo^†^, Yuanqing Sun^†^, Juqi Chai, Lei Liu, Jiaqi Li, Yuekun Ren, Changhong Guo***

The following supplemental materials is available for this article:

**
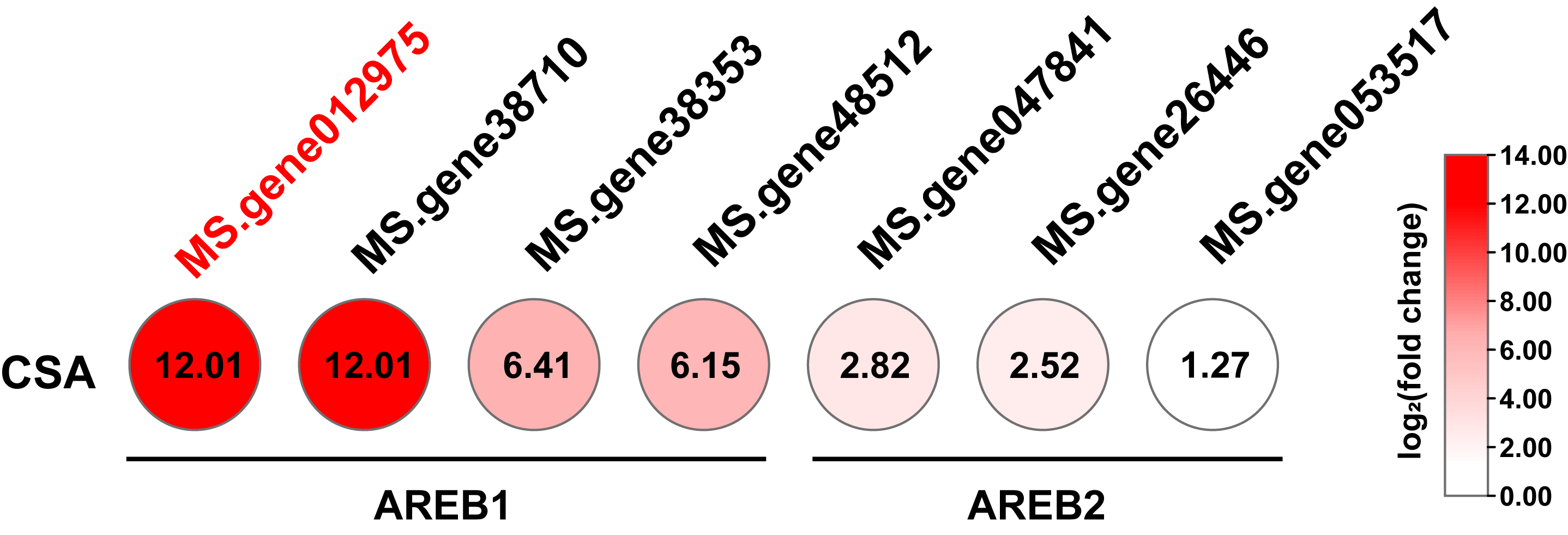
**

**Figure S1** Heat map of AREB TFs derived from transcriptome data analysis. The fragments per kilobase of transcript per million mapped reads (FPKM) values were taken as a measure of gene expression, and the values were represented after normalization of log_2_FPKM.

**
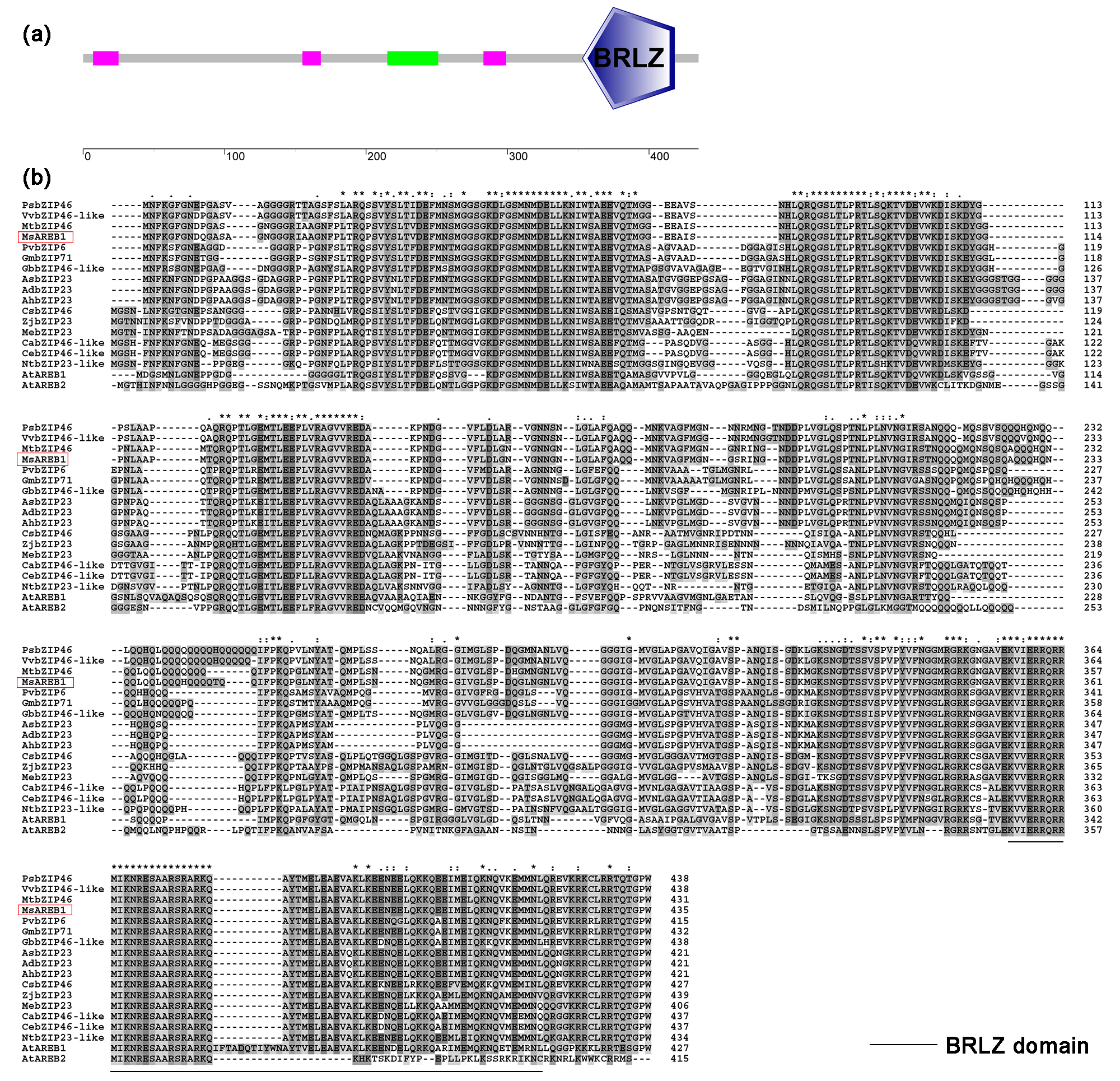
**

**Figure S2** Conserved domain and sequence analyses of MsAREB1. (a) Analysis of conserved domains of MsAREB1. (b) Sequence alignment of the amino acids of MsAREB1 with other plant AREB/bZIPs. Accession numbers: MsAREB1 (MS.gene012975.t1), PsbZIP46 (XP_050885000.1), VvbZIP46-like (XP_058786200.1), GbbZIP46-like (XP_061343227.1), PvbZIP6 (AAK39132.1), GmbZIP71 (NP_001341123.1), AsbZIP23 (XP_057728236.1), AdbZIP23 (XP_015934248.1), AhbZIP23 (XP_025612611.1) CsbZIP46 (XP_030499075.1), NtbZIP23-like (XP_009592273.1), MebZIP23 (XP_021601955.1), CabZIP46-like (XP_027094182.1), CebZIP46-like (XP_027148287.1), ZjbZIP23 (XP_015884345.3), MtBZIP46 (XP_003603049.1), AtAREB1 (NP_001185157.1), and AtABRE2 (NP_001154626.1)

**
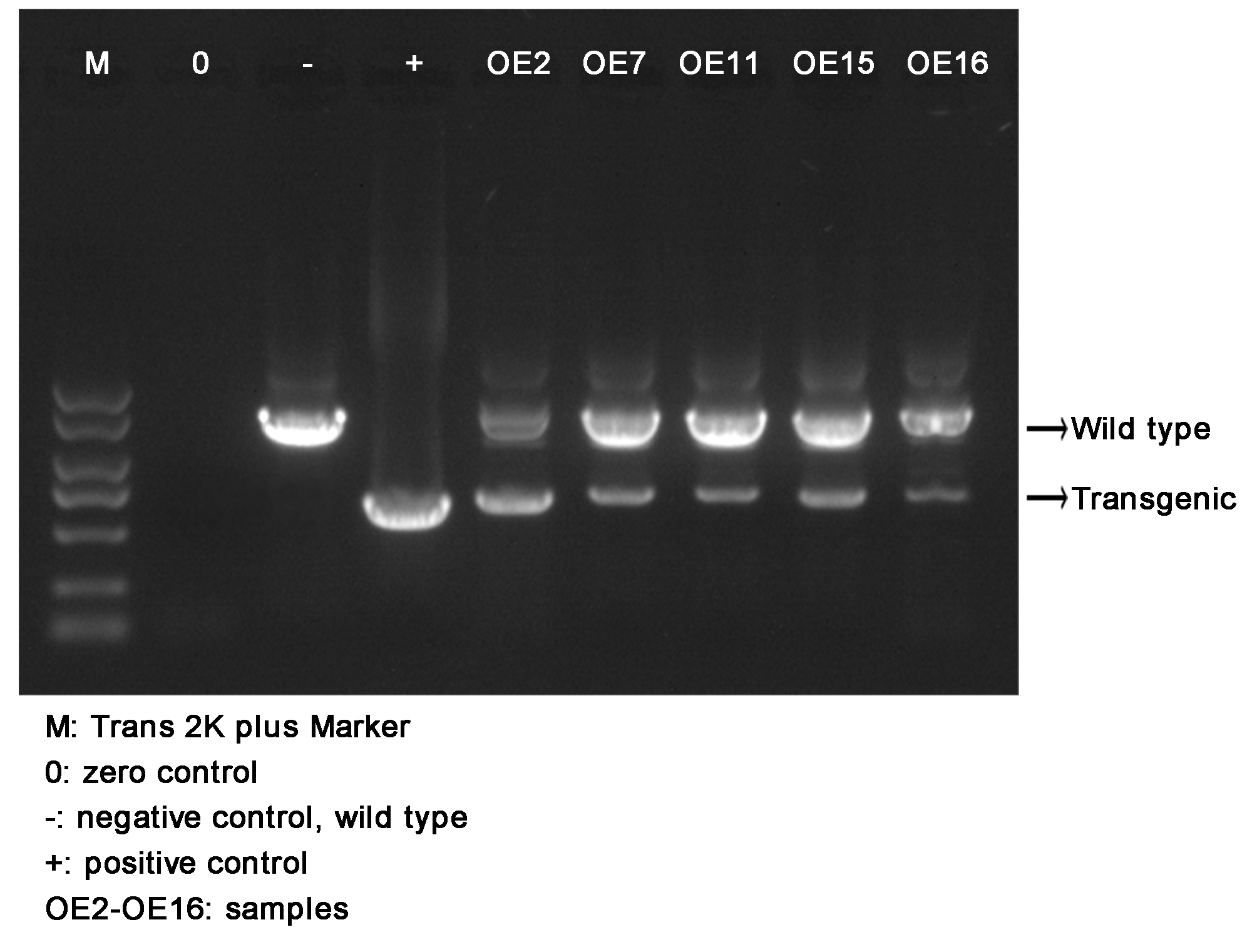
**

**Figure S3** The DNA identification of *MsAREB1* gene in *MsAREB1*-OE transgenic alfalfa. OE2, OE7, OE11, OE15, and OE16 represent five independent *MsAREB1*-OE lines.

**
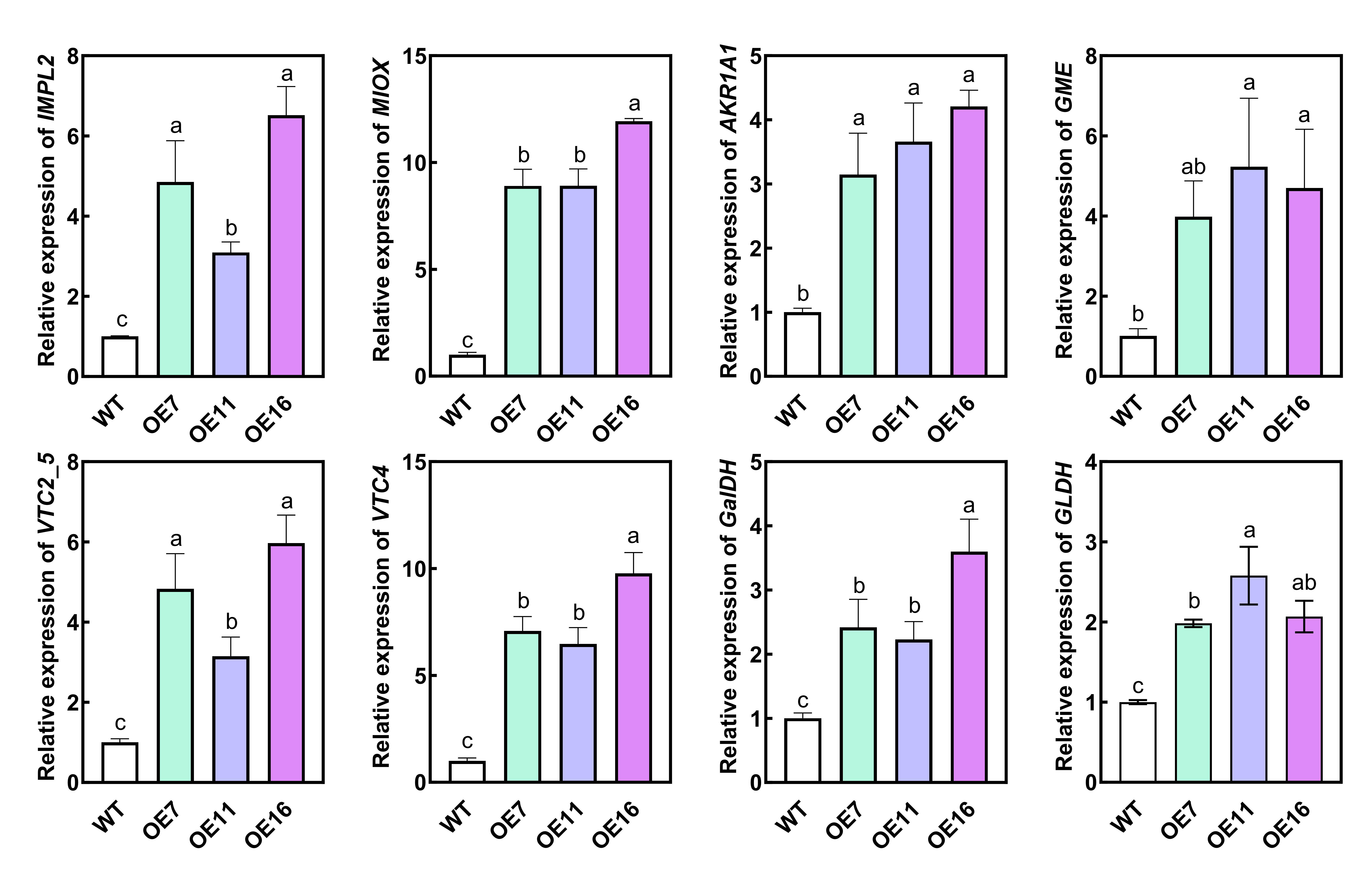
**

**Figure S4** The expression of key genes involved in AsA biosynthesis in WT and *MsAREB1*-OE transgenic alfalfa. Accession numbers: *IMPL2* (MS.gen58789.t1), *MIOX* (MS.gen58966.t1), *AKR1A1* (MS.gen073524.t1), *GME* (MS.gen44007.t1), *VTC2_5* (MS.gen027017.t1), *VTC4* (MS.gen047283.t1), *GALDH* (MS.gen90275.t1), and *GDH* (MS.gen073666.t1). OE7, OE11, and OE16 represent three independent *MsAREB1*-OE lines. *MsActin* was used as an internal standard. Data are means ± SE (*n* = 3). Bars with different letters indicate significant difference at *P* < 0.05.

**
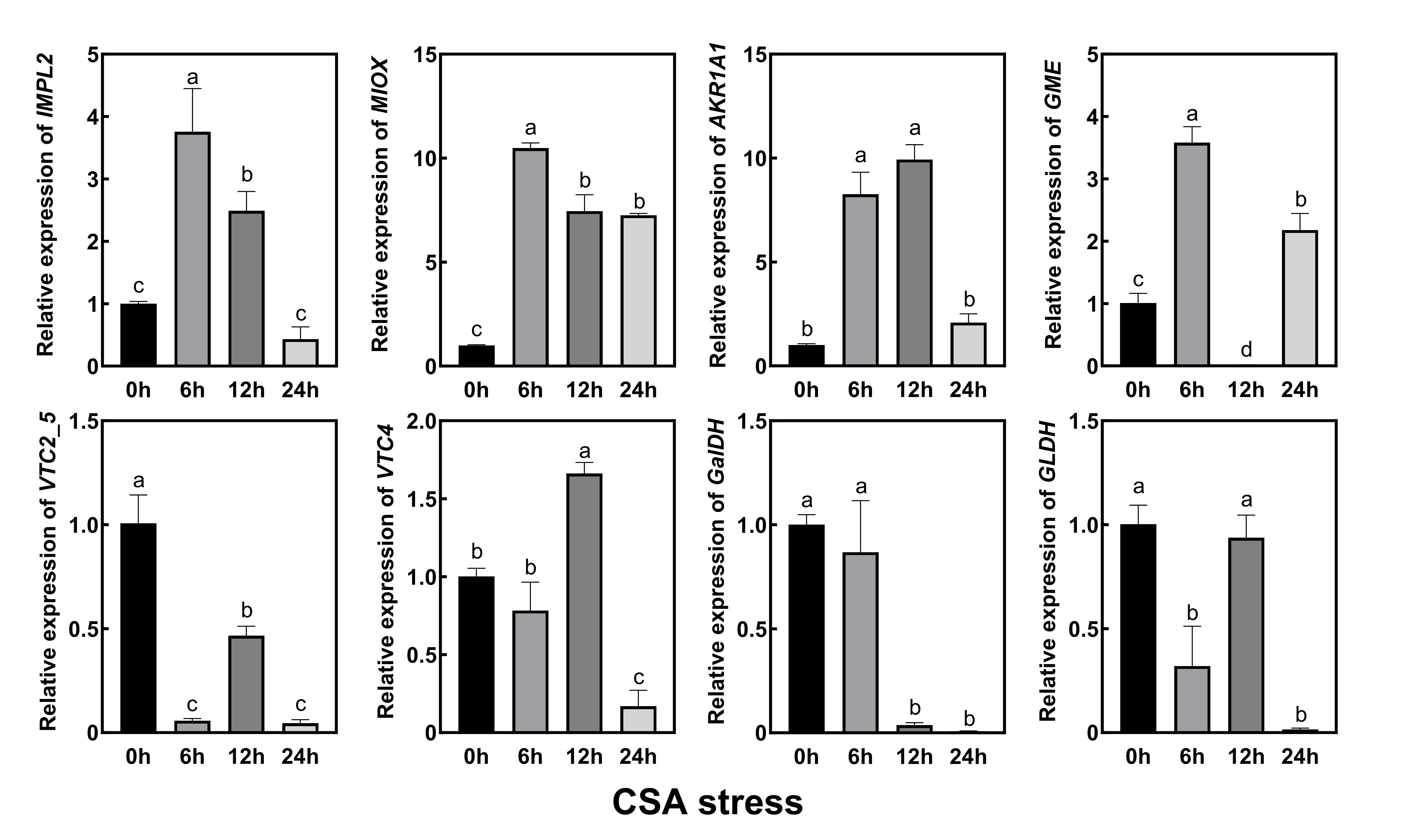
**

**Figure S5** The expression of key genes involved in AsA biosynthesis in CSA stress treated alfalfa. Samples were collected at 6 h, 12 h, and 24 h after the initiation of the stress, samples collected at 0 h were non-treated control. *MsActin* was used as an internal standard. Data are means ± SE (*n* = 3). Bars with different letters indicate significant difference at *P* < 0.05.

**
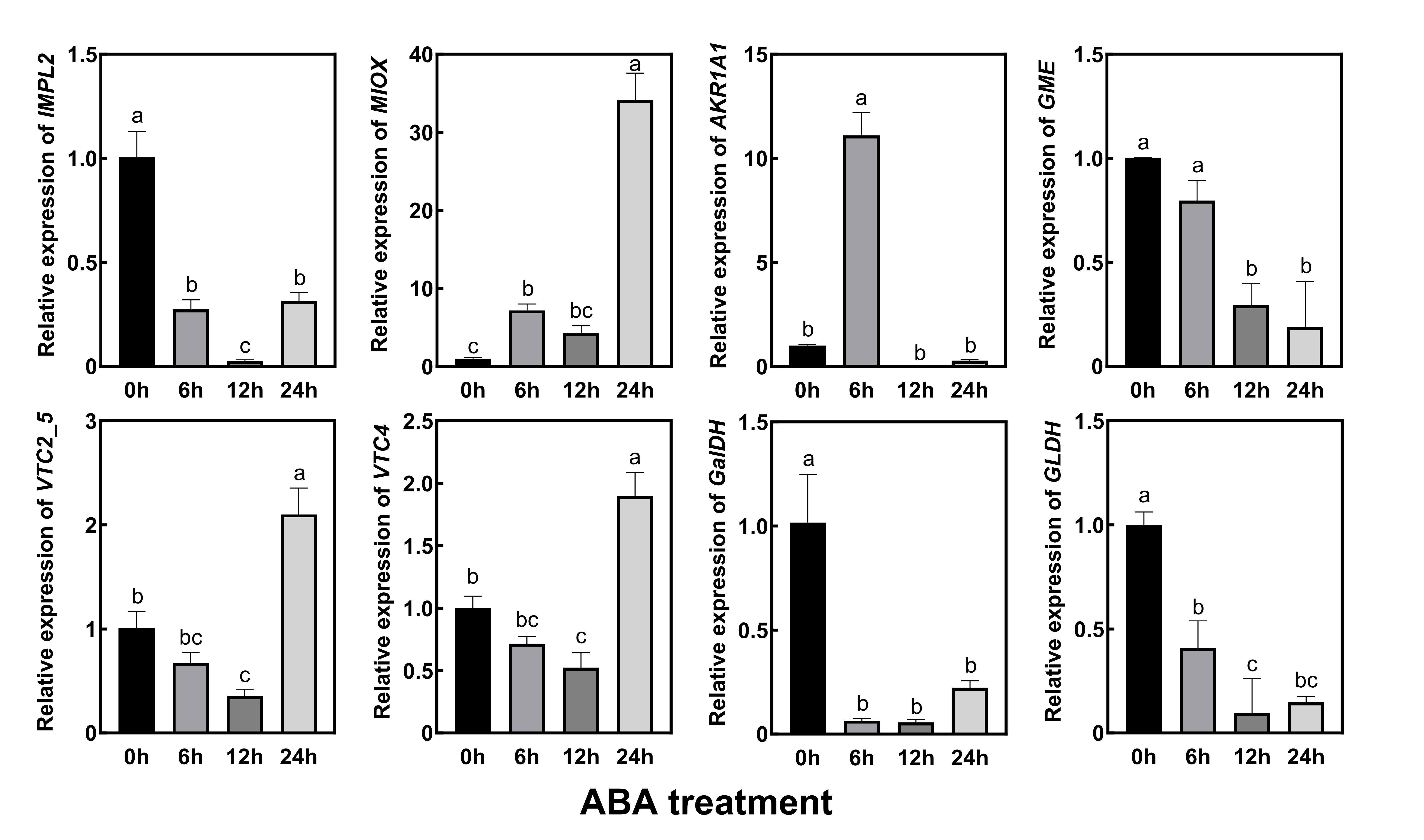
**

**Figure S6** The expression of key genes involved in AsA biosynthesis in ABA solution treated alfalfa. Samples were collected at 6 h, 12 h, and 24 h after the initiation of the treatment, samples collected at 0 h were non-treated control. *MsActin* was used as an internal standard. Data are means ± SE (*n* = 3). Bars with different letters indicate significant difference at *P* < 0.05.

**
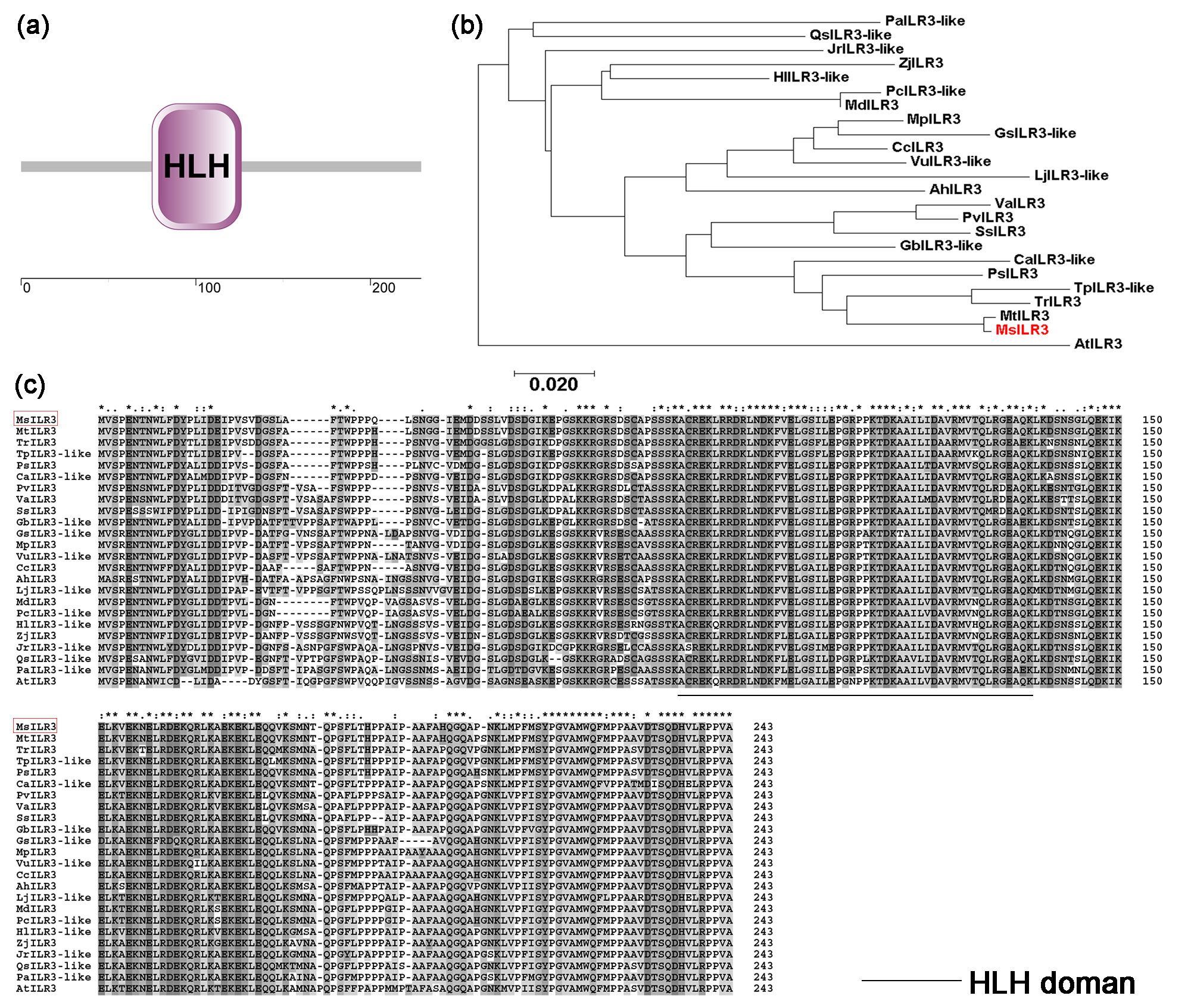
**

**Figure S7** Conserved domain, phylogenetic, and sequence analyses of MsILR3. (a) Analysis of conserved domains of MsILR3. (b) Neighbour-joining (NJ) tree of MsILR3. Bootstrap values (1000 replicates) are indicated in the branches. (c) Sequence alignment of the amino acids of MsILR3 with other plant ILR3s. Accession numbers: MsILR3 (MS.gene031275.t1), AtILR3 (Q9FH37.1), MtILR3 (XP_003596613.1), TrILR3 (KAK2423993.1), TpILR3-like (XP_045802062.1), GbILR3-like (XP_061341785.1), CaILR3-like (XP_004487616.1), PvILR3 (XP_068499907.1), AhILR3 (XP_025687925.1), PsILR3 (XP_050883217.1), VuILR3-like (XP_027910857.1), MdILR3 (XP_008365724.1), PcILR3-like (XP_068311040.1), GsILR3-like (XP_028194508.1), QsILR3-like (KAJ7948495.1), SsILR3 (TKY48987.1), LjILR3-like (XP_057457131.1), CcILR3 (XP_020229330.1), MpILR3 (RDX72117.1), JrILR3-like (XP_018852414.1), HlILR3-like (XP_062077823.1), VaILR3 (XP_017425015.1), PaILR3-like (XP_028761774.1), and ZjILR3 (XP_015898140.2).

**
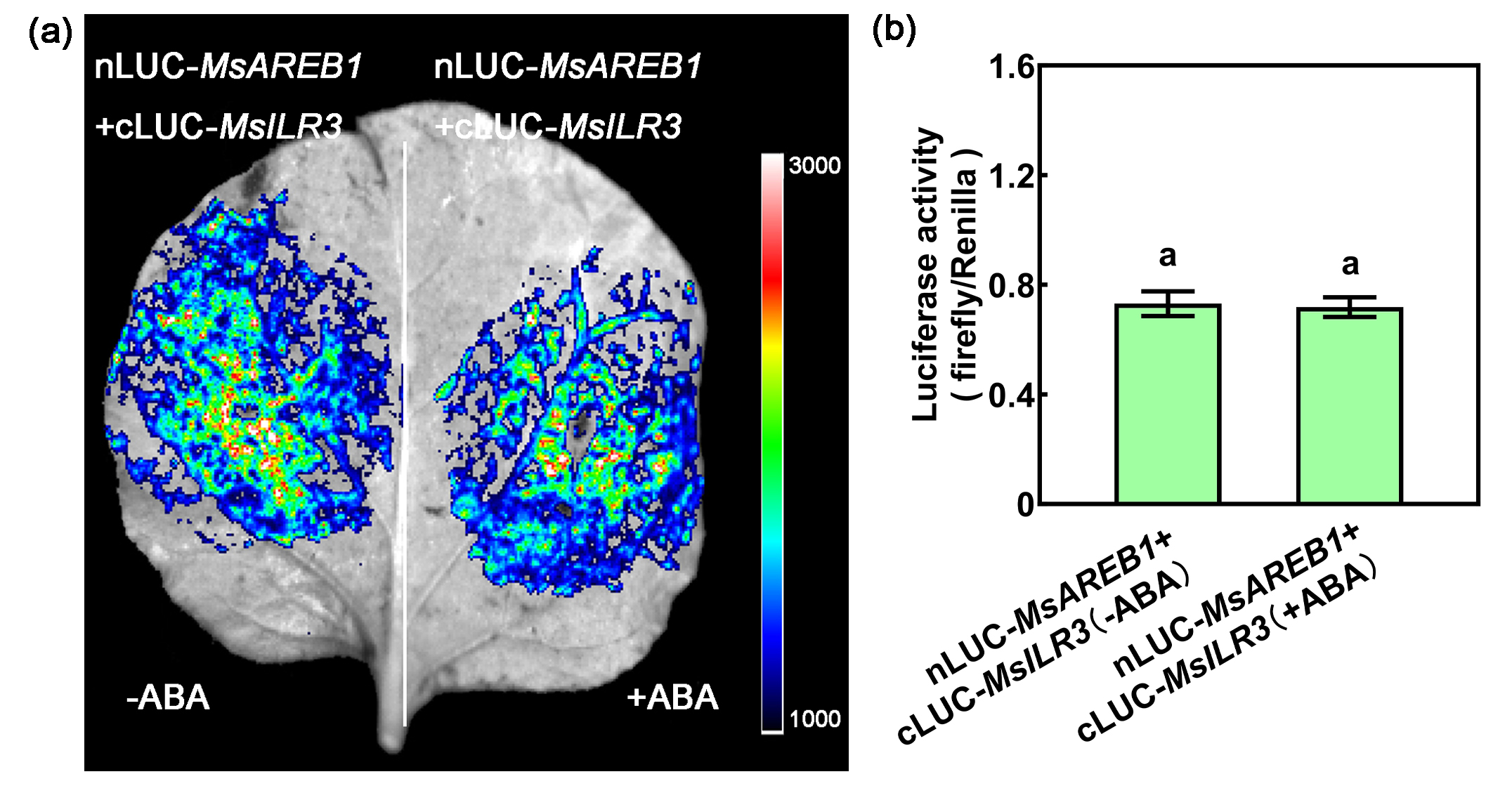
**

**Figure S8** LCI assay using nLUC-MsAREB1 and cLUC-MsILR3 constructs; *Agrobacterium* cultures were combined at a 1 : 1 (v/v) ± 2 μM ABA, then infiltrated into *Nicotiana benthamiana* leaves. Data are means ± SE (*n* = 3). Bars with different letters indicate significant difference at *P* < 0.05.


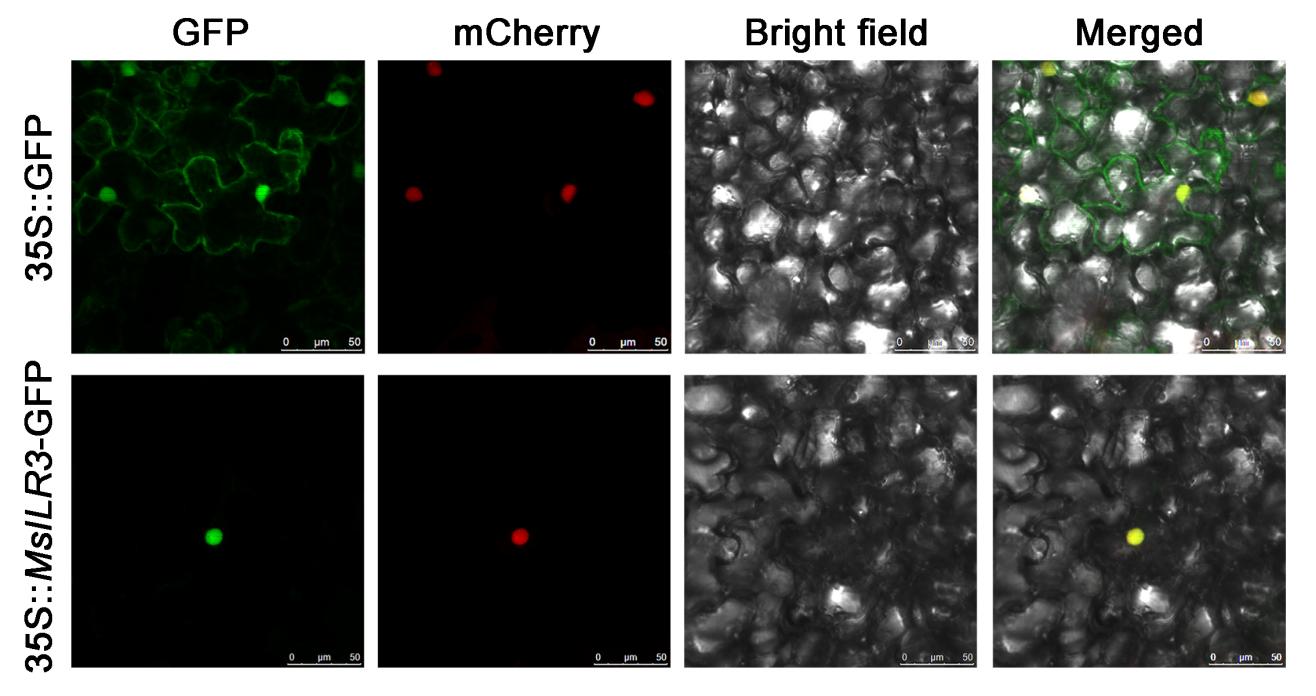


**Figure S9** Subcellular localization of the 35S::*MsILR3*-GFP fusion protein in *Nicotiana benthamiana* leaf epidermal cells. The mCherry protein indicates nucleus localization. Scale bars: 50 μm.

**
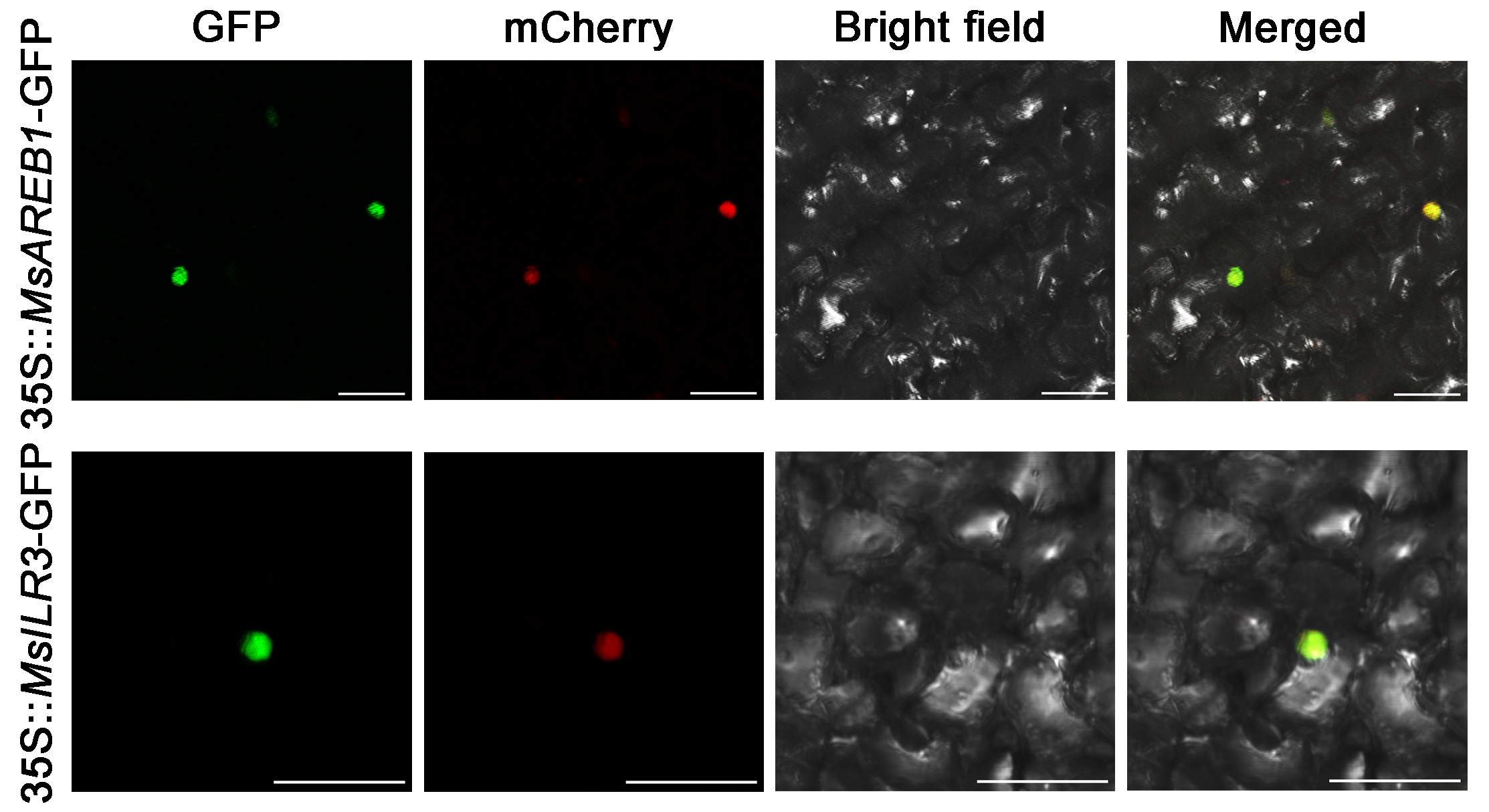
**

**Figure S10** Subcellular localization of the 35S::*MsAREB1*-GFP and 35S::*MsILR3*-GFP fusion protein in *Nicotiana benthamiana* leaf epidermal cells under CSA stress. The mCherry protein indicates nucleus localization. Scale bars: 50 μm.

**
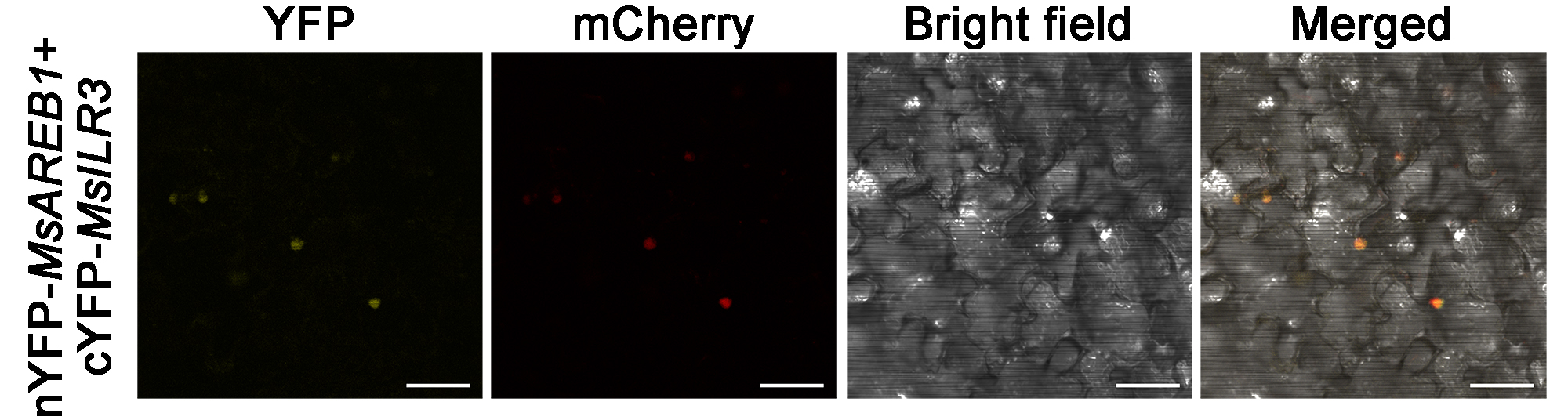
**

**Figure S11** BiFC assay infers the interaction between MsAREB1 and MsILR3 in *Nicotiana benthamiana* leaves under CSA stess. The mCherry protein indicates nucleus localization. Scale bars: 50 μm.

**Table S1** Primers used in this study.

| **Primer name** | **Sequence 5' to 3'** |
| --- | --- |
| **Primers for gene and promoter cloning** | |
| MsAREB1-F | ATGAATTTCAAGGGTTTTG |
| MsAREB1-R | CTACCATGGACCAGTTTG |
| MsILR3-F | ATGGTTTCCCCGGAAAAC |
| MsILR3-R | TTAGGCAACTGGTGGACGG |
| **Primers for constructs used in the subcellular localization experiment** | |
| eGFP-MsAREB1-F | TAGCTCTAGA**GGATCC**ATGAATTTCAAGGGT |
| eGFP-MsAREB1-R | TGCTCACCAT**GGATCC**CCATGGACCAGTTTG |
| eGFP-MsILR3-F | TAGCTCTAGA**GGATCC**ATGGTTTCCCCGGAAAAC |
| eGFP-MsILR3-R | TGCTCACCAT**GGATCC**GGCAACTGGTGGACGG |
| **Primers for constructs used to generate transgenic plants** | |
| pCAMBIA1302-MsAREB1-F | GGACTCTTGA**CCATGG**ATGAATTTCAAGGGTTTTG |
| pCAMBIA1302-MsAREB1-R | GTCAGATCTAC**CCATGG**CCATGGACCAGTTTG |
| **Primers used to identify the transgenic alfalfa** | |
| OE-MsAREB1(cross intron)-F | CTCAGCAGATGAATAAAGTT |
| OE-MsAREB1(cross intron)-R | CTCTTCTTTTAACTTTGCGA |
| **Primers for constructs used in the Y1H assay** | |
| pGADT7-MsAREB1-F | GGAGGCCAGT**GAATTC**ATGAATTTCAAGGGT |
| pGADT7-MsAREB1-R | CACCCGGGTG**GAATTC**CCATGGACCAGTTTG |
| pGADT7-MsILR3-F | GGAGGCCAGT**GAATTC**ATGGTTTCCCCGGAAAAC |
| pGADT7-MsILR3-R | CACCCGGGTG**GAATTC**GGCAACTGGTGGACGG |
| pAbAi-MsMIOX2pro(3×G-Box)-F | GCTTGAATTC**GAGCTC**CACGTGCACGTGCACGTG**GTCGAC**CTCGAGGCA |
| pAbAi-MsMIOX2pro(3×G-Box)-R | TGCCTCGAG**GTCGAC**CTCATGCTCATGCTCATG**GAGCTC**GAATTCAAGC |
| **Primers for the** **Dual-LUC assay** | |
| pCAMBIA1302-MsILR3-F | GGACTCTTGA**CCATGG**ATGGTTTCCCCGGAA |
| pCAMBIA1302-MsILR3-R | GTCAGATCTAC**CCATGG**GGCAACTGGTGGACG |
| pGreenⅡ0800-MsMIOX2pro-F | GTCGACGGTATCGAT**AAGCTT**CTAGTAAAAGCAGAAACA |
| pGreenⅡ0800-MsMIOX2pro-R | CGCTCTAGAACTAGT**GGATCC**GTGAAATGAGTATGGAAT |
| **Primers for constructs used in the EMSA assay** | |
| pCold-MsAREB1-F | ATTCAAGCTT**GTCGAC**ATGAATTTCAAGGGT |
| pCold-MsAREB1-R | TAGACTGCAG**GTCGAC**CCATGGACCAGTTTG |
| pCold-MsILR3-F | ATTCAAGCTT**GTCGAC**ATGGTTTCCCCGGAA |
| pCold-MsILR3-R | TAGACTGCAG**GTCGAC**GGCAACTGGTGGACG |
| Biotin-(MsMIOX2pro)G-Box probe-F | TGGCAACTGC**CACGTG**TGCTTCAAAA |
| Biotin-(mMsMIOX2pro)G-Box probe-F | TGGCAACTGC**TTCATG**TGCTTCAAAA |
| Unlabeled-(MsMIOX2pro)G-Box probe-R | TTTTGAAGCA**CACGTG**GCAGTTGCCA |
| **Primers used to generate DNA constructs for the Y2H assay** | |
| pGBKT7-MsAREB1-F | CATGGAGGCC**GAATTC**ATGAATTTCAAGGGT |
| pGBKT7-MsAREB1-R | GGATCCCCGG**GAATTC**CCATGGACCAGTTTG |
| pGBKT7-MsAREB1-C-F | CATGGAGGCC**GAATTC**GTAATGGAGCTGTGG |
| pGBKT7-MsAREB1-C-R | GGATCCCCGG**GAATTC**CCATGGACCAGTTTG |
| pGBKT7-MsAREB1-N-F | CATGGAGGCC**GAATTC**ATGAATTTCAAGGGT |
| pGBKT7-MsAREB1-N-R | GGATCCCCGG**GAATTC**CTCCACAGCTCCATT |
| **Primers for constructs used in the BiFc assay** | |
| pCAMBIA1300-nYFP-MsAREB1-F | CGCCACTAGT**GGATCC**ATGAATTTCAAGGGT |
| pCAMBIA1300-nYFP-MsAREB1-R | TACTATCGAT**GGATCC**CCATGGACCAGTTTG |
| pCAMBIA1300-cYFP-MsILR3-F | CGCCACTAGT**GGATCC**ATGGTTTCCCCGGAA |
| pCAMBIA1300-cYFP-MsILR3-R | TACTATCGAT**GGATCC**GGCAACTGGTGGACG |
| **Primers for constructs used in the LCI assay** | |
| pCAMBIA1300-nLUC-MsAREB1-F | GGACGAGCTC**GGTACC**ATGAATTTCAAGGGT |
| pCAMBIA1300-nLUC-MsAREB1-R | CGTACGAGATCTG**GTCGAC**CCATGGACCAGTTTG |
| pCAMBIA1300-cLUC-MsILR3-F | GTCCCGGGGC**GGTACC**ATGGTTTCCCCGGAA |
| pCAMBIA1300-cLUC-MsILR3-R | TGTTGCTGCAG**GTCGAC**GGCAACTGGTGGACG |
| **Primers for constructs used in the Pull-down assay** | |
| pGEX-4T-1-MsILR3-F | TGGATCCCCG**GAATTC**ATGGTTTCCCCGGAA |
| pGEX-4T-1-MsILR3-R | GTCGACCCGG**GAATTC**GGCAACTGGTGGACG |
| **Primers for analyzing genes expression** | |
| MsActin-Q-F | CAAAAGATGGCAGATGCTGAGGAT |
| MsActin-Q-R | CATGCACCAGTATGACGAGGTCG |
| MsAREB1-Q-F | AAAGGTAATGGAGCTGTGGA |
| MsAREB1-Q-R | GCATTTTCTCTTGACTTCTCG |
| MsILR3-Q-F | GCGGTATTGAAATGGATG |
| MsILR3-Q-R | ATTGCTGCCTTGTCTGTT |
| IMPL2-Q-F | CCTCATCTTCGCCTCCTC |
| IMPL2-Q-R | TACCCAAACATAATCAGCAC |
| MIOX-Q-F | TAGAACCTTGCTTTTGCTGTAG |
| MIOX-Q-R | CAATGTGATGATGTCATGGGG |
| AKR1A1-Q-F | TATTCTCCTTTAGGCTCACC |
| AKR1A1-Q-R | CCAGAGTTCTTCAATGGTCCT |
| GME-Q-F | TGTCGGAAAGCAATCACG |
| GME-Q-R | CTCCCTTTCAAGTTGTTCCT |
| VTC2_5-Q-F | GGTTCCTACGGTTGTTTCG |
| VTC2_5-Q-R | TTTCACAGGCAGTAACATCAT |
| VTC4-Q-F | TTCAAGCCAAAGCCACG |
| VTC4-Q-R | GCTCCGATTCACTGTTTCC |
| GalDH-Q-F | GGCGATGGGTCTTCTCAC |
| GalDH-Q-R | TGACAGGCTTCAGAATGGTT |
| GLDH-Q-F | GCCAAGGGGACAATAGAAG |
| GLDH-Q-R | CTCACAACCACGGCAGAC |
